# Strong hyperconjugative interactions limit solvent and substituent influence on conformational equilibrium: the case of *cis*-2-halocyclohexylamines

**DOI:** 10.3762/bjoc.15.79

**Published:** 2019-04-01

**Authors:** Camila B Francisco, Cleverton S Fernandes, Ulisses Z de Melo, Roberto Rittner, Gisele F Gauze, Ernani A Basso

**Affiliations:** 1 Chemistry Department, State University of Maringá, 5790, Maringá 87020-900, Brazil; 2 Chemistry Institute, University of Campinas, 6154, Campinas 13083-970, Brazil

**Keywords:** conformational equilibrium, cyclohexane derivatives, dynamic NMR, hyperconjugation, principal component analysis

## Abstract

The presence of strong stereoelectronic interactions involving the substituents in *cis*-2-substituted cyclohexanes may lead to results different from those expected. In this work, we studied the conformational behavior of *cis*-2-fluoro- (**F**), *cis*-2-chloro- (**Cl**), *cis*-2-bromo- (**Br**) and *cis*-2-iodocyclohexylamine (**I**) by dynamic NMR and theoretical calculations. The experimental data pointed to an equilibrium strongly shifted toward the **ea** conformer (equatorial amine group and axial halogen), with populations greater than 90% for **F**, **Cl** and **Br** in both dichloromethane-*d*_2_ and methanol-*d*_4_. Theoretical calculations (M06-2X/6-311++G(2df,2p)) were in agreement with the experimental, with no influence of the solvent or the halogen on the equilibrium. A principal component analysis of natural bond orbital energies pointed to the σ*_C–X_ and σ_C–H_ orbitals and the halogen lone pairs (LP_X_) as the most significant for the hyperconjugative interactions that influenced the equilibrium. The σ_C–H_ → σ*_C–X_ hyperconjugation and the interactions involving the LP_X_ counterbalance each other, explaining the non-influence of the halogen on the conformational equilibrium. These interactions are responsible for the strong preference for the **ea** conformer in *cis*-2-halocyclohexylamines, being strong enough to restrain the shift in the equilibrium due to other factors such as steric repulsion or solvent effects.

## Introduction

*cis*-2-Substituted cyclohexanes are interesting from the conformational point of view, since one of the substituents should be axial. Generally, bulky substituents prefer the equatorial position to avoid steric repulsions [1]. Nevertheless, two distinct groups may provoke a competition of interactions, with regard to their volumes and steric repulsions, and the particular effects of the solvation medium on them, besides the intrinsic stereoelectronic effects. The sum of these factors in a system may lead to results different from those expected by considering just simple generalizations [2–9].

In the case of *cis*-2-halocyclohexylamines, the balance of interactions occurs between an amine group, which is known to be sensitive to the solvent [2,10–11], and the halogen, where the size increases considerably from F to I.

Batchelor reported the solvent effects on *cis*-2-methylcyclohexylamine [11], where the variation from aprotic to protic solvent causes a significant shift in the equilibrium, changing the axial preference of the amine group, which becomes equatorial with the protonation of the nitrogen.

The variation in the halogen size is also expected to cause some effect on the conformational equilibrium. Freitas and co-workers have demonstred this effect on *trans*-1,2-dihalocyclohexanes [12], where the diequatorial repulsion between the bulky halogens make the diaxial conformer more stable. Similarly, in *trans*-2-halocyclohexanols the OH–X interaction that stabilizes the diequatorial conformer loses strength for steric repulsions in going from F to I [13]. However, in the behavior observed by Basso and co-workers for *cis*-2-halocyclohexanols [14], the axial preference of the halogen (about 60–70%) is not greatly affected by the increase in the halogen size. Instead, the equilibrium is influenced by solute–solvent hydrogen bonding with the hydroxy group.

Besides the classical effects, interactions such as hyperconjugation has been pointed out as relevant in several studies involving cyclohexane derivatives [4,12,15–20]. Since the positions of both bonding and antibonding orbitals of the substituents change with the ring inversion, a conformation where a given substituent favors these interactions by performing them more effectively, may, in fact, govern the equilibrium. The C–X bonds have already been reported as excellent electron-density acceptors, and their presence in a system suggests the possibility of these interactions [17,21].

Although there have been several works on the conformational preference of 1,2-disubstituted cyclohexanes [4,11–14,20,22–29], *cis*-2-halocyclohexylamines have not yet been consistently studied. This is quite surprising, due to the possibilities of effects that add up or compete with each other in the presence of these two groups. This gap in the literature may be attributed to experimental difficulties in obtaining these derivatives.

**Figure 1:**
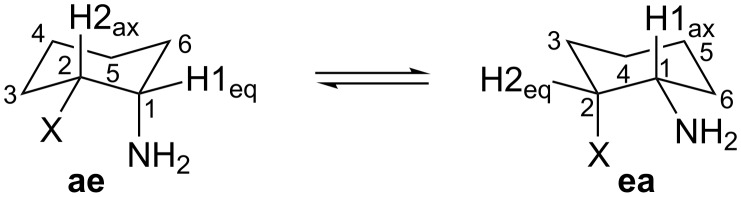
Conformations of *cis*-2-halocyclohexylamines, where X = F, Cl, Br and I.

Therefore, in this work, we developed the study of *cis*-2-fluorocyclohexylamine (**F**), *cis*-2-chlorocyclohexylamine (**Cl**), *cis*-2-bromocyclohexylamine (**Br**) and *cis*-2-iodocyclohexylamine (**I**), the latter being only theoretical (due to experimental difficulties). We evaluated the conformational equilibrium between **ae** (axial amine group and equatorial halogen) and **ea** (equatorial amine group and axial halogen) conformers (Figure 1) by dynamic NMR (DNMR) and theoretical calculations.

## Results and Discussion

### Experimental conformational population

**Figure 2:**
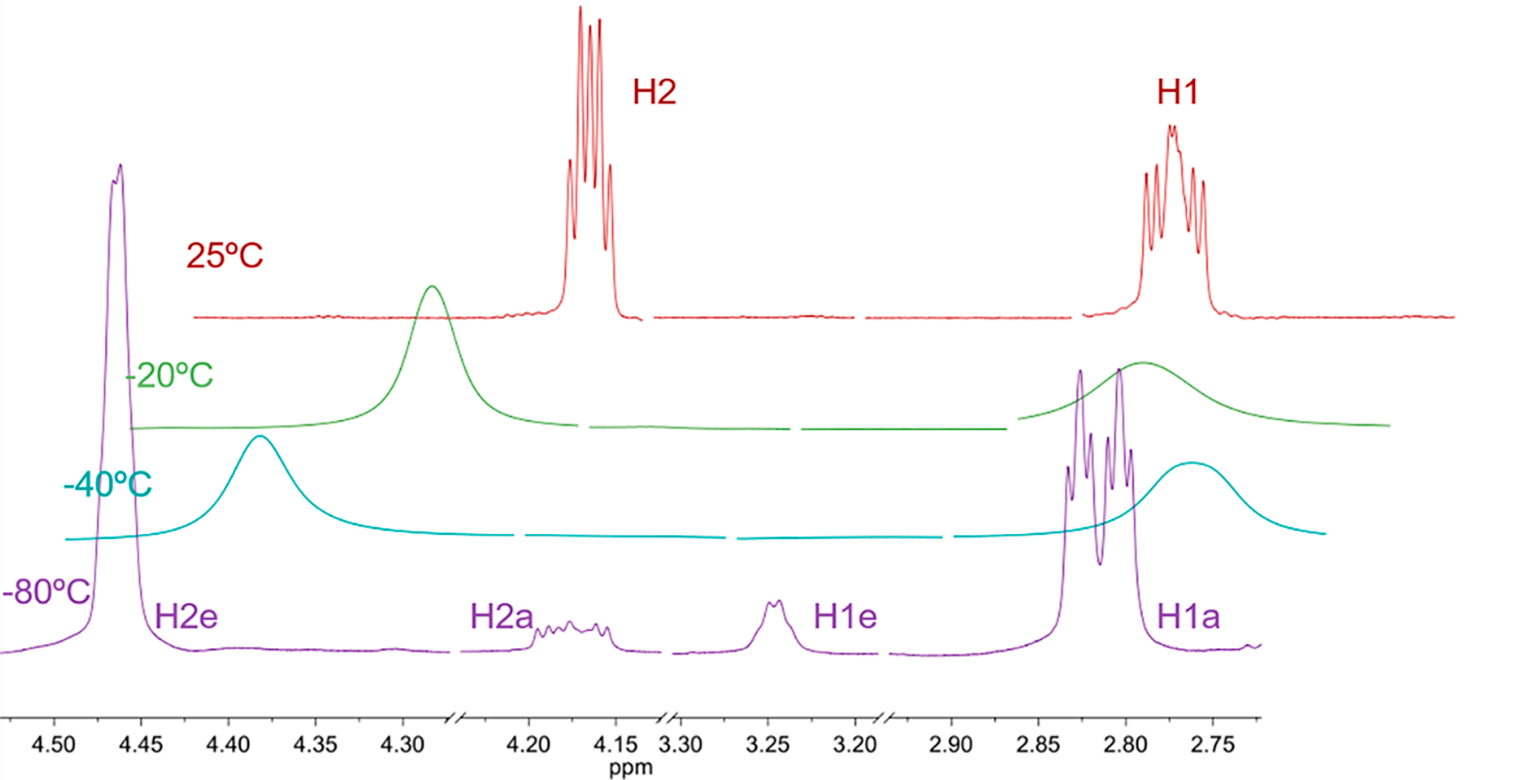
Variable-temperature ^1^H NMR spectra (500.13 MHz) for *cis*-2-chlorocyclohexylamine in dichloromethane-*d*_2_.

Low-temperature NMR experiments allow the identification of the individual conformers and their population at equilibrium, determined through the integration of ^1^H and ^13^C NMR spectra at −80 °C. Figure 2 illustrates the changes in the ^1^H NMR spectra of *cis*-2-chlorocyclohexylamine in dichloromethane-*d*_2_ by varying the temperature from 25 to −80 °C.

At 25 ºC, the signal at 2.89 ppm corresponds to H1, vicinal to nitrogen, and the signal at 4.32 ppm to H2, vicinal to chlorine (Figure 2). These ^1^H resonances, at room temperature, correspond to the average of the conformers at equilibrium. Table 1 shows the spectral data for **F**, **Cl** and **Br** at 25 and −80 °C, where ^3^*J*_H–H_ refers to the individual coupling constants with the vicinal hydrogens, and W is the sum of all couplings, the latter being useful when there is loss of definition or signal broadening, common in low-temperature experiments [29].

**Table 1:** Chemical shifts (δ, ppm), coupling constants (^3^*J*_H–H_, Hz) and half-height line width (W, Hz) for *cis*-2-halocyclohexylamines in different solvents at 25 °C and −80 °C.

		Hydrogen	δ^a^	^3^*J*_H-H_*^b^*	W		δ^a^	^3^*J*_H-H_^b^	W		δ^a^	^3^*J*_H-H_^b^	W
			
			Fluorine				Chlorine				Bromine		
					
dichloromethane-*d*_2_	25 °C	H1	2.73	10.21^c^	19.21		2.89	8.96	19.42		2.97	9.81	17.83
		H2	4.60	4.74^c^	11.36		4.32	5.72	12.99		4.68	5.65	11.10
	−80 °C	H1_ax_	2.62	11.86^c^	22.76		2.81	11.48	20.61		3.03	^d^	^d^
		H1_eq_	3.37	^d^	16.89		3.25	3.33	10.54		3.52	^d^	^d^
		H2_ax_	4.55	^d^	23.18		4.17	10.69	22.46		4.80	^d^	^d^
		H2_eq_	4.72	^d^	10.16		4.46	^d^	10.15		4.88	^d^	^d^

methanol-*d*_4_	25 °C	H1	2.70	10.83^c^	19.32		2.86	9.48	19.45		2.90	^d^	18.53
		H2	4.64	4.40^c^	10.59		4.37	5.40	12.18		4.64	3.61	10.51
	−80 °C	H1_ax_	2.58	12.01^c^	20.59		2.78	11.51	20.73		2.97	^d^	^d^
		H1_eq_	3.21	^d^	12.52		3.10	3.31	11.32		3.32	^d^	^d^
		H2_ax_	4.48	^d^	23.11		4.19	12.16	22.47		4.76	^d^	^d^
		H2_eq_	4.65	2.56^c^	8.10		4.41	^d^	8.10		4.67	^d^	^d^

^a^In relation to TMS as reference. ^b^Largest H–H coupling constant at three bonds. ^c^Measured considering only the ^3^*J*_H–H_ coupling, disregarding ^2^*J*_H–F_. ^d^*J* and W were not measured due to loss of resolution or signals broadening at −80 °C.

According to the coupling constants (^3^*J*_H–H_) and half-height line widths (W) measured at 25 °C, the dominant conformation is **ea**. The higher values of *J* and W for H1 indicate the diaxial coupling of this hydrogen with the axial hydrogen of C6 (Figure 1). This behavior was observed for all compounds, in both solvents, as shown in Table 1.

At −80 °C, two signals for H1 and H2 are observed, which correspond to **ae** (H1_ax_ and H2_eq_) and **ea** (H1_eq_ and H2_ax_) conformers individually (Table 1). The values of ^3^*J*_H–H_, δ and W at −80 °C allow the assignment of the signals to the respective conformers, and by the integrals, determination the percentage of each in the equilibrium.

In the case of the bromine derivative, due to the broadening of both ^1^H and ^13^C signals and the loss of signal resolution at low temperature, the assignments at −80 °C were made by considering the attributions at 25 °C. So, we assume the **ea** conformer to be the major, attributing the most intense signals set at −80 °C to this conformer.

The populations determined by the integration of both ^1^H and ^13^C NMR spectra at −80 °C and the relative conformational energies are shown in Table 2. The equilibrium is shifted strongly toward the **ea** conformer, with 90% population for **F** and **Cl** and 91% for **Br** in dichloromethane-*d*_2_, while in methanol-*d*_4_ the populations were 95, 94 and 91% for **F**, **Cl** and **Br**, respectively. These results clearly show that the equilibrium is non-sensitive to halogen size and solvent effects.

**Table 2:** Conformational populations and relative conformational energies of *cis*-2-halocyclohexylamines at −80 °C in different solvents.

	^1^H			^13^C			Average	
					
	% **ea**	Δ*G*º^a^		% **ea**	Δ*G*º^a^		% **ea**	Δ*G*º^a^

	**F**							

dichloromethane-*d*_2_	90	−0.84		90	−0.84		90	−0.84
methanol-*d*_4_	95	−1.13		96	−1.22		95	−1.13

	**Cl**							

dichloromethane-*d*_2_	90	−0.84		90	−0.84		90	−0.84
methanol-*d*_4_	93	−0.99		95	−1.13		94	−1.06

	**Br**							

dichloromethane-*d*_2_	91	−0.89		^b^	–		–	–
methanol-*d*_4_	91	−0.89		^b^	–		–	–

^a^Δ*G* = − RT ln *K*, where *K* = *n*_ea_/*n*_ae_ being *n*_ea_ the population of **ea** conformer and *n*_ae_ the population of **ae** conformer. A negative value means that **ea** is more stable. ^b 13^C integration was not possible due to signal broadening.

A similar axial preference of cyclohexane halo derivatives was observed by Basso and co-workers in *cis*-2-halocyclohexanols [14], and by de Oliveira and Rittner in *trans*-3-halocyclohexanols [30]. However, in both studies this preference was not so pronounced as that observed in this work, with a small difference between the populations of the two conformers at equilibrium.

The equatorial preference for the amine group was somehow expected, due to classical steric effects (*syn*-1,3-diaxial repulsion). On the other hand, the amine group is usually very sensitive to the solvent effect. In addition, the increase in the halogen size could lead to a shift toward the **ae** conformer (equatorial halogen). In the studied system, no change was observed, and this behavior suggests that other effects can be acting. So, theoretical calculations were performed in order to understand which mechanisms control the conformational preference of these compounds.

### Theoretical study

#### Conformer energy

**Figure 3:**
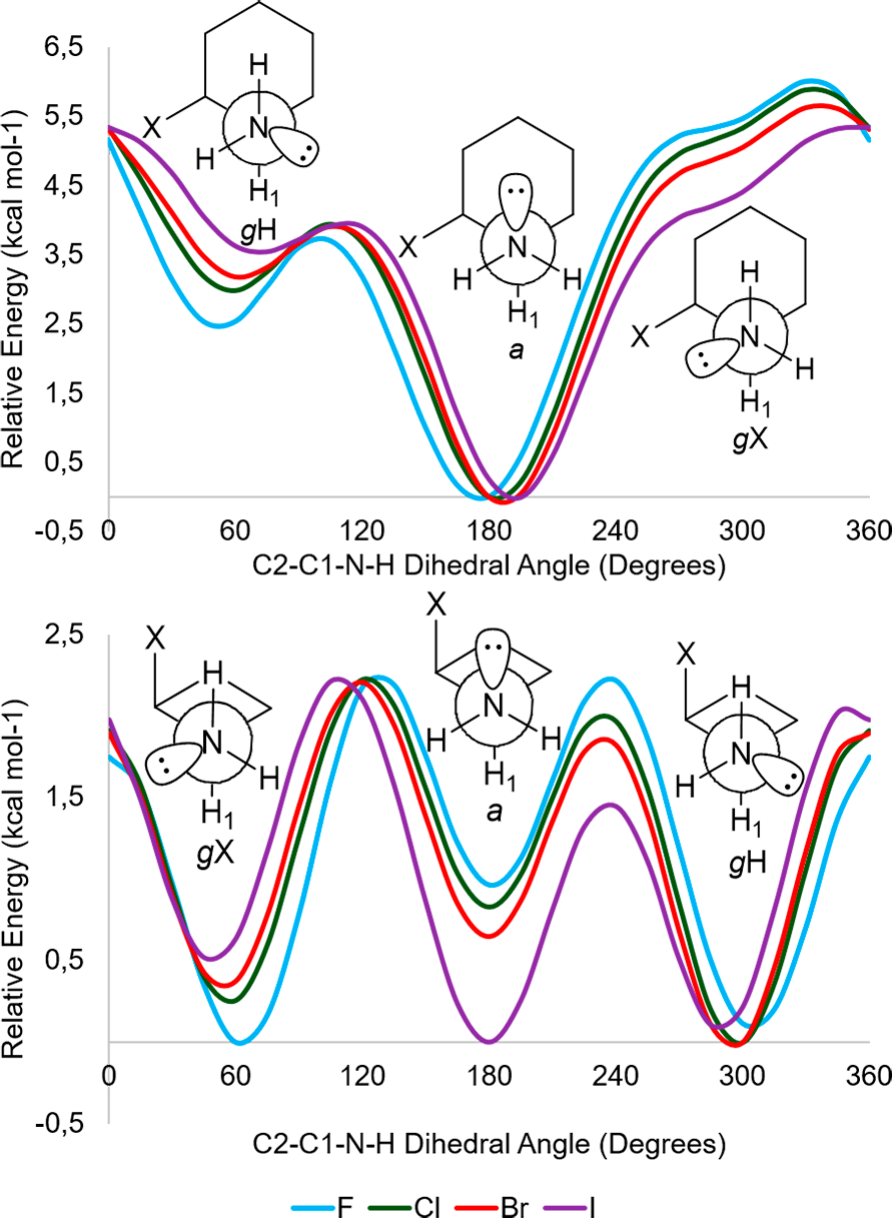
Potential energy surfaces (PESs) for *cis*-2-halocyclohexylamines for the C2–C1–N–H dihedral angle rotation at M06-2X/aug-cc-pVDZ in **ae** (top) and **ea** (bottom) conformers.

The potential energy surface (PES) for the dihedral C2–C1–N–H (M06-2X/aug-cc-pVDZ) shows that each conformer has three lower-energy rotamers, identified according to the orientation of the nitrogen lone pair as anti (*a*) and gauche (*g*X and *g*H) to H1 (Figure 3).

Optimization and frequency calculations were performed for these rotamers at different theory levels. A table showing the energies of each rotamer is available in Supporting Information File 1 (Table B1).

We compiled in Table 3 the energy differences between **ea** and **ae** conformers (Δ*E*_ea_), at four different theory levels, together with the theoretical **ea** populations. The Δ*E*_ea_ values were calculated by the weighted averages of both conformers, considering the rotational populations in each. The negative values, both in gas phase and in solution, indicate that **ea** is the more stable conformation.

**Table 3:** Variation of conformational energy (Δ*E*_ea_, kcal mol^−1^) and populations of **ea** conformer determined theoretically in *cis*-2-halocyclohexylamines.

		Gas phase			Dichloromethane			Methanol	
					
		Δ*E*_ea_	% **ea**		Δ*E*_ea_	% **ea**		Δ*E*_ea_	% **ea**

M06-2X/aug-cc-pVDZ	F	−0.32	74		−0.69	88		−0.66	86
	Cl	−0.46	79		−0.66	86		−0.70	88
	Br	−0.49	81		−0.89	92		−0.88	92
	I	−0.48	81		−1.02	94		−1.06	94
RMSD^a^					0.131			0.270	

M06-2X/6-311++G(2df,2p)	F	−0.36	76		−0.67	86		−0.69	87
	Cl	−0.49	80		−0.76	89		−0.77	90
	Br	−0.50	81		−0.93	92		−0.93	92
	I	−0.36	76		−0.95	92		−0.98	93
RMSD^a^					0.004			0.230	

MP2/aug-cc-pVDZ	F	−0.22	71		−0.61	78		−0.73	88
	Cl	−0.22	70		−0.64	85		−0.68	87
	Br	−0.40	79		−0.74	81		−0.87	92
	I	−0.86	92		−1.47	98		−1.50	98
RMSD^a^					0.010			0.234	

MP2/6-311++G(2df,2p)	F	−0.16	68		−0.68	86		−0.68	87
	Cl	−0.22	70		−0.68	86		−0.72	88
	Br	−0.17	67		−0.64	85		−0.62	85
	I	−0.16	66		−0.63	85		−0.63	86
RMSD^a^					0.013			0.264	

^a^Root mean square deviation of experimental and theoretical Δ*G*º, being the last available in Supporting Information File 1 (Table B2).

A trend in energy values was observed at all the theory levels employed in this study, there being a small decrease of Δ*E*_ea_ from gas phase to solution. However, when we compare the Δ*E*_ea_ values for dichloromethane and methanol, the energy variation is too small (Table 3). Likewise, the nature of the halogen also showed a minimal variation in Δ*E*_ea_ values over the series, both in gas phase and in solution. These energetical reports are in agreement with the experimental.

The calculations performed in M06-2X/6-311++G(2df,2p) showed results closer to the experimental, with the lower root mean square deviation (RMSD) error values of 0.004 in dichloromethane and 0.230 in methanol. This shows the efficiency of this theory level to describe the studied systems. The agreement of the energy values in gas phase with those in solution confers reliability to calculations such as natural bond orbitals (NBOs), which are made in the gas phase.

**Figure 4:**
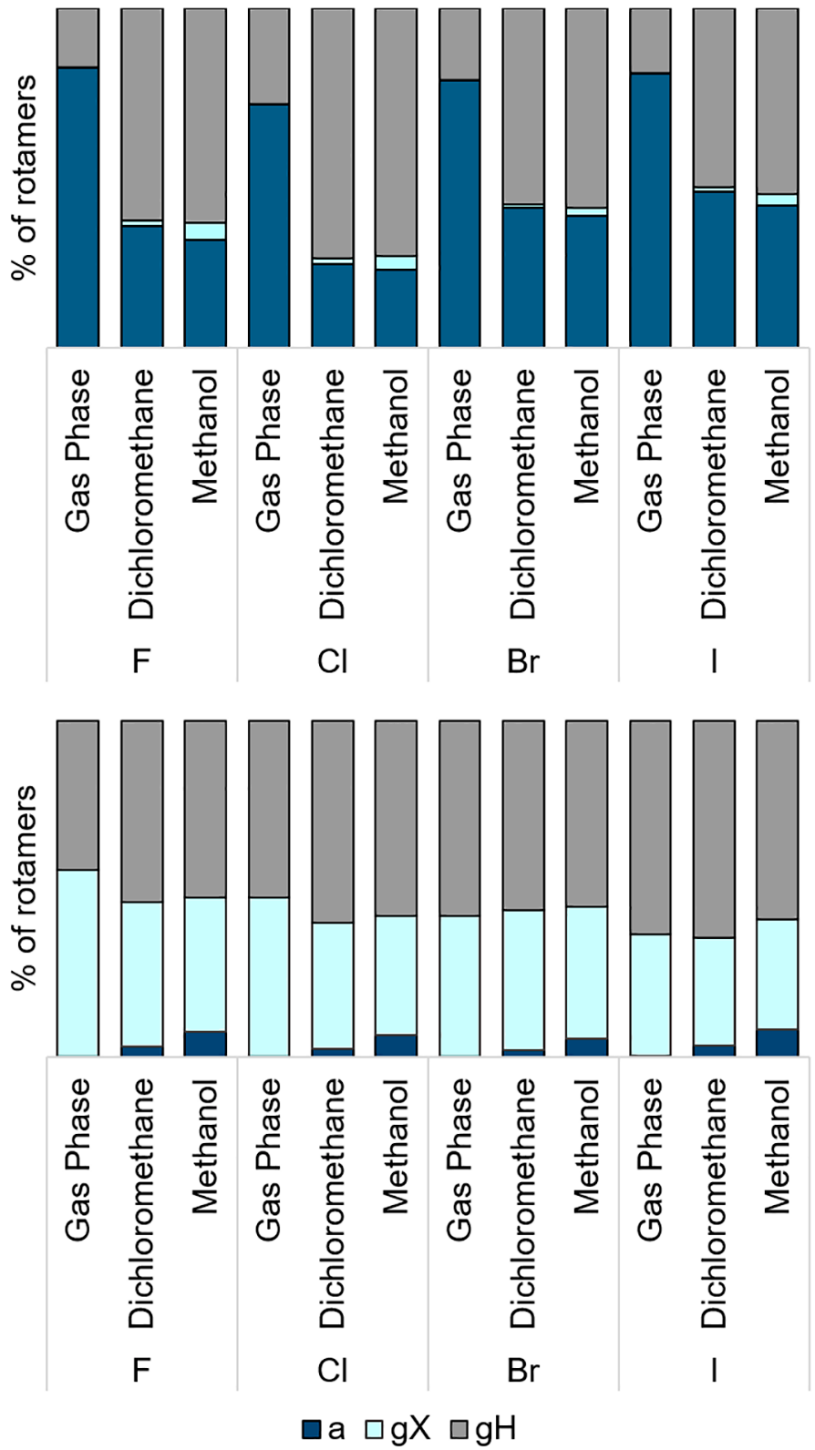
Populations of rotamers *a*, *g*X e *g*H in **ae** (top) and **ea** (bottom) conformers of *cis*-2-halocyclohexylamines in the gas phase, dichloromethane and methanol.

With regard to rotamers, *g*X in **ae** conformer and *a* in **ea** are the least stable, with smaller populations (calculated in M06-2X/6-311++G(2df,2p)), as shown in Figure 4. This was observed for the calculations performed in the gas phase, and in dichloromethane and methanol.

Even though previous works involving cyclohexane halo derivatives reported the axial preference of halogens, as mentioned before, the pronounced **ea** preference observed in our case led us to investigate the specific effects that may be responsible for this behavior.

#### Delocalization, electrostatic and steric interactions

In order to determine which effects are responsible for the stabilization of each conformer, we carried out studies of hyperconjugative, steric and electrostatic effects.

The deletion of all non-Lewis natural bond orbitals (M06-2X/6-311++G,2df,2p) disregard all hyperconjugative effects, allowing us to evaluate the conformational preference in the absence of these interactions. Therefore, the total energy of each conformer (*E*_total_) can be expressed as the sum of *E*_loc_, the localized energy after the deletion of the hyperconjugation, and *E*_deloc_, the delocalization energy [31].


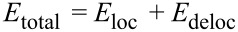


These calculations also provide the total steric exchange energy (*E*_steric_), which describes the electron cloud repulsion [32]. Another parameter evaluated was the total electrostatic energy, *E*_elect_, according molecular mechanics calculations employing the Amber force field GAFF [33].

So, after the deletion of hyperconjugative interactions, we should analyze the delocalization energy, where higher values indicate stronger effects. In Table 4 only the most stable rotamers for each conformation were analyzed. The values of Δ*E*_deloc_ show an inversion of the conformational preference for all compounds in the absence of hyperconjugative interactions, and **ae** becoming the most stable conformer. It is worth noting the increase in the difference of the delocalization energy along the series, suggesting the influence of the halogen in this effect.

**Table 4:** Variation in total (Δ*E*_total_), localized (Δ*E*_loc_) and delocalization (Δ*E*_deloc_) energies; and absolute total steric exchange (*E*_steric_) and total electrostatic (*E*_elect_) energies, in kcal mol^−1^, for *cis*-2-halocyclohexylamines.

	Δ*E*_total_^a^	Δ*E*_loc_^a^	Δ*E*_deloc_^a^	*E*_steric_^a^	*E*_elect_^b^

	Fluorine				

**ae**	0.25	0.00	3.46	507.05	−0.50
**ea**	0.00	3.21	0.00	509.24	−0.22

	Chlorine				

**ae**	0.25	0.00	5.77	527.16	−0.13
**ea**	0.00	5.52	0.00	532.02	0.18

	Bromine				

**ae**	0.32	0.00	7.56	534.05	−0.09
**ea**	0.00	7.24	0.00	535.73	0.25

	Iodine				

**ae**	0.40	0.00	8.18	514.35	−0.04
**ea**	0.00	7.78	0.00	518.27	0.31

^a^M06-2X/6-311++G(2df,2p) in the gas phase. ^b^Calculated using the Amber force field GAFF. Positive values indicate more repulsive interactions.

Taking into account the localized energy (Δ*E*_loc_), the **ea** conformer presented the highest values, and it is consistent with the values of *E*_steric_ and *E*_elect_. It shows that, although **ea** is the most stable conformation, it has the greater steric and electrostatic repulsions.

Analyzing all these effects together, we could observe that hyperconjugation is an important factor to describe the conformational preference. Despite the evident influence of the halogen on hyperconjugation, the balance of these energies reflects in the unchanged equilibrium along the series.

**Figure 5:**
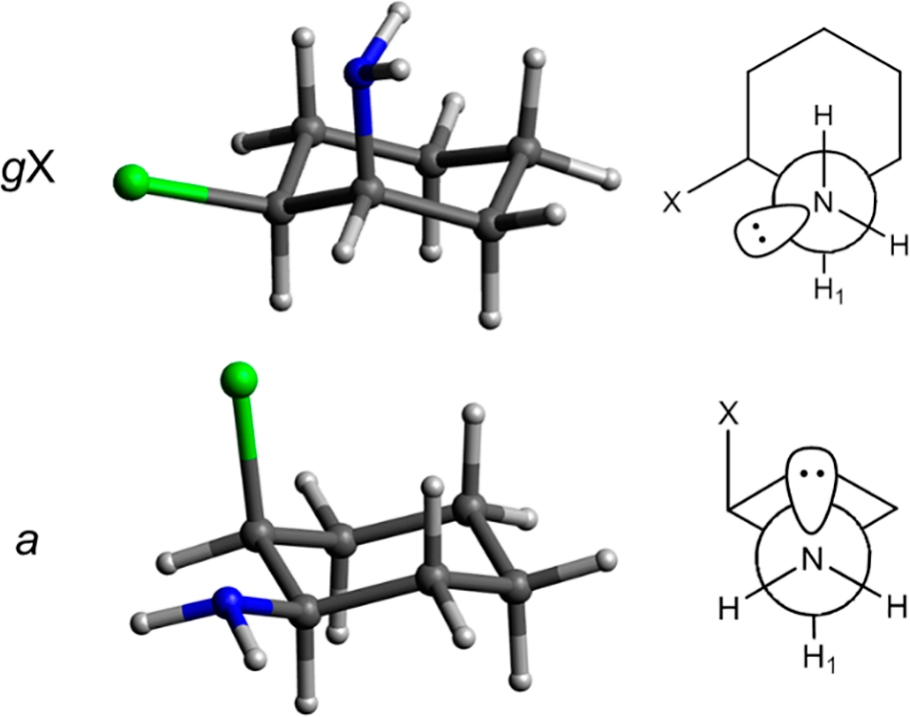
Nitrogen lone pair in rotamers *g*X (**ae**) and *a* (**ea**) oriented towards the halogen, making these rotamers less stable in their respective conformation.

With respect to the rotamers, neither hyperconjugation nor total steric exchange energies can explain the rotational behavior, which is attributed to electrostatic effects only. In the most unstable rotamers of each conformer (*g*X for **ae** and *a* for **ea**) the nitrogen electron lone pair is oriented toward the halogen, as illustrated in Figure 5, resulting in greater electrostatic repulsions. The values of localized, delocalized, steric and electrostatic energies for all rotamers are available in Supporting Information File 1 (Table B3).

Although there is a sum of effects that can govern a conformational equilibrium, for *cis*-2-halocyclohexylamines the rotational preference is due to electrostatic effects, while the strong and non-sensitive conformational preference for **ea** can be explained by hyperconjugation. This arouses the interest for a deeper analysis of hyperconjugative interactions.

#### Principal component analysis (PCA) of natural bond orbitals (NBOs)

In order to interpretate the large number of interactions present in the compound structures, we applied PCA to NBO energies.

For each individual bond, the sum was made of all hyperconjugative stabilization energies (*E_ij_*) involving both bonding and antibonding orbitals, as well as the energies of nitrogen and halogen lone-pair interactions (LP_N_ and LP_X_, respectively), resulting in 22 variables corresponding to all the bonds of the structure of the most stable rotamer in each conformation (see Supporting Information File 2 for full numerical data).

**Figure 6:**
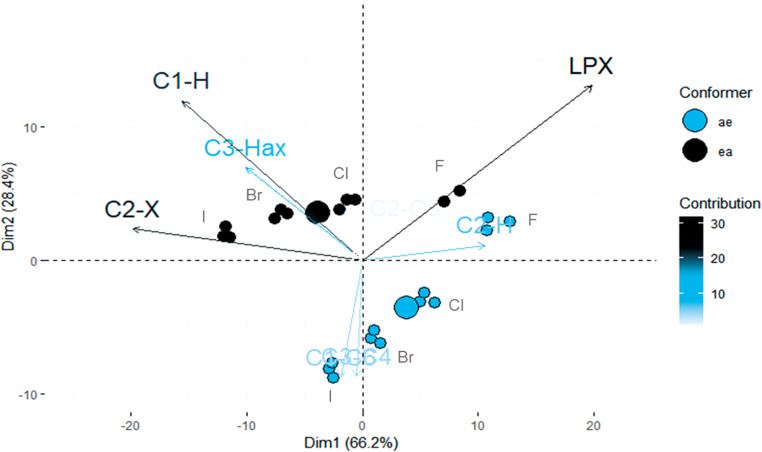
PCA for 22 variables corresponding to the hyperconjugative interactions for all rotamers in **ae** and **ea** conformers of *cis*-2-halocyclohexylamines. Note that the correlation vectors with the strongest black color has the greater contribution for the PC’s.

In the PCA (Figure 6), two principal components described 94% of the results. The PC1 is able to differentiate the variables according to the halogens, while the PC2 distinguish the two conformations.

The fact of the PC1, which has the higher variance in relation to the others, distinguish the halogens following a chemical trend is quite important, since the change in the halogen and consequently in the size of the orbitals directly affects the electronic delocalization.

According to the correlation vectors, the LP_X_ differentiate the variables between the halogens, with the greater weight for the fluoro derivatives. If we analyse the vectors perpendicular to the groups of conformers, it is clear that the variables corresponding to C1–H, C2–X and C3–H_ax_ bonds distinguish the **ea** conformer from **ae**.

Then, considering all the interactions existent in both conformations, the ones involving the LP_X_ are important for both conformers. However, what differs them are the interactions with the highest weight for **ea**.

It is worth mentioning that the variables corresponding to the N–H bonds and LP_N_ has no contribution for the PC’s, confirming that the rotational preference has no influence on the hyperconjugation.

**Figure 7:**
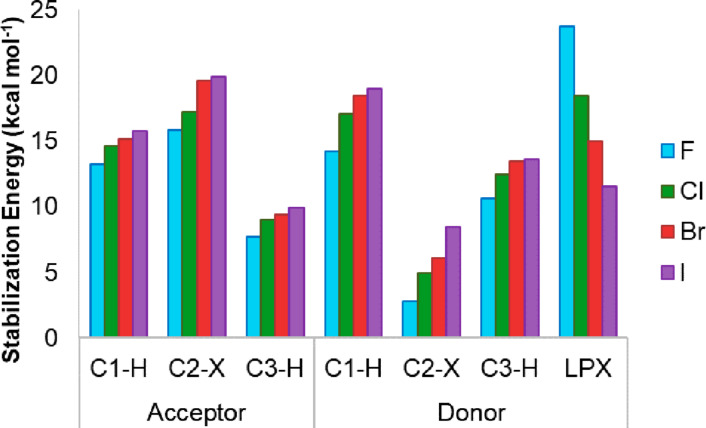
Sum of bonding (donor) and antibonding (acceptor) orbitals interactions of C1–H, C2–X and C3–H_ax_ bonds and LP_X_ in *cis*-2-halocyclohexylamines.

Analyzing the bonds pointed by the PCA as electron density donors (bonding orbitals) or acceptors (antibonding orbitals) in Figure 7, it is clear that the most significant interactions are those involving the C2–X bond acting as electronic density acceptor (σ*_C2–X_ orbital), and the C1–H (σ_C1–H_ orbital) and LP_X_ acting as donor.

This shows that hyperconjugative interactions are dependent on the halogen: as the size of the halogen increases, the donor or acceptor ability of the respective orbitals (C–X bonds and antibonds) increases in the same way; however, the LP_X_ follows the reverse order, with a decrease in the donor ability as LP_F_ > LP_Cl_ > LP_Br_ > LP_I_.

These observations allow us to analyze which specific orbitals are responsible for the great stabilization of the **ea** conformer. Table 5 presents the stabilization energy values (*E_ij_*), the difference in energy between the orbitals (*E_i_* − *E_j_*) and the Fock matrix elements (*F*_(_*_i_*_:_*_j_*_)_) for the main hyperconjugative interactions.

**Table 5:** Stabilization energy, *E_ij_* (kcal mol^−1^), orbitals energy difference *E_i_* − *E_j_* (a. u.) and Fock matrix elements *F*_(_*_i_*_;_*_j_*_)_ (a. u.) of main hyperconjugative interactions in **ea** conformer of *cis*-2-halocyclohexylamines.

	*E_ij_*	*E_i_* - *E_j_*	*F*_(_*_i_*_;_*_j_*_)_

σ_C1–H_ → σ*_C2–X_			

**F**	5.83	0.86	0.063
**Cl**	7.88	0.72	0.067
**Br**	9.13	0.66	0.069
**I**	9.44	0.64	0.069

σ_C3–Hax_ → σ*_C2–X_			

**F**	5.99	0.87	0.065
**Cl**	7.43	0.73	0.066
**Br**	8.39	0.67	0.067
**I**	8.34	0.65	0.066

LP_X_ → σ*_C–C_^a^			

**F**	6.09	0.94	0.068
**Cl**	4.36	0.79	0.053
**Br**	3.40	0.76	0.046
**I**	2.52	0.72	0.038

LP_X_ → σ*_C2–H8_			

**F**	6.26	0.97	0.069
**Cl**	4.52	0.86	0.056
**Br**	3.19	0.85	0.046
**I**	2.15	0.81	0.037

^a^σ*_C2–C3_ for **F**, and σ*_C1–C2_ for **Cl**, **Br** and **I**.

The *E_ij_* values for σ_C1–H_ → σ*_C2–X_ and σ_C3–Hax_ → σ*_C2–X_ clearly show a trend from **F** to **I,** where these interactions become considerably more intense (higher *E_ij_* values) with the increase in the halogen size. It is also possible to observe that the difference in energy between these orbitals (*E_i_* − *E_j_*) decreases from **F** to **I**, which means that the acceptance of electron density by the C–X bond is more effective when we have the larger halogens. The *E_i_* − *E_j_* values decrease from 0.86 for **F** to 0.64 atomic units for **I** in σ_C1–H_ → σ*_C2–X_; and from 0.87 for **F** to 0.65 atomic units for **I** in σ_C3–Hax_ → σ*_C2–X_. The same trend is observed for the Fock matrix element (*F*_(_*_i_*_:_*_j_*_)_), which concerns to the orbital overlap and also increases from **F** to **I** .

On the other hand, the interactions involving the LP_X_ follow the reverse order (Table 5). The *E_ij_* values decrease steadily from **F** to **I**, going from 6.09 and 6.26 to 2.52 and 2.15 kcal mol^−1^ for LP_X_ → σ*_C–C_ and LP_X_ → σ*_C2–H8_, respectively. In the same way, the Fock matrix element decreases considerably comparing the LP_F_ and LP_I_ interactions, going from 0.068 and 0.069 for LP_F_ to 0.038 and 0.037 atomic units for LP_I_, in LP_X_ → σ*_C–C_ and LP_X_ → σ*_C2–H8_, respectively.

This tendency in the halogen series observed in both hyperconjugative interactions describes the observed in PCA; the LP_X_ interactions counterbalance the σ_C–H_ → σ*_C2–X_, in the sense that the LP_X_ is more important for the fluoro derivatives, while the σ_C–H_ → σ*_C2–X_ has the greater weight for the larger halogens.

The acceptor ability of σ*_C–X_ orbitals has already been described in the literature [17], and has an inverse correlation with electronegativity: the electronegativity decreases from **F** to **I** and the acceptor ability of σ*_C–X_ orbitals increases in the order σ*_C–F_ < σ*_C–Cl_ < σ*_C–Br_ < σ*_C–I_, as observed in this work.

**Figure 8:**
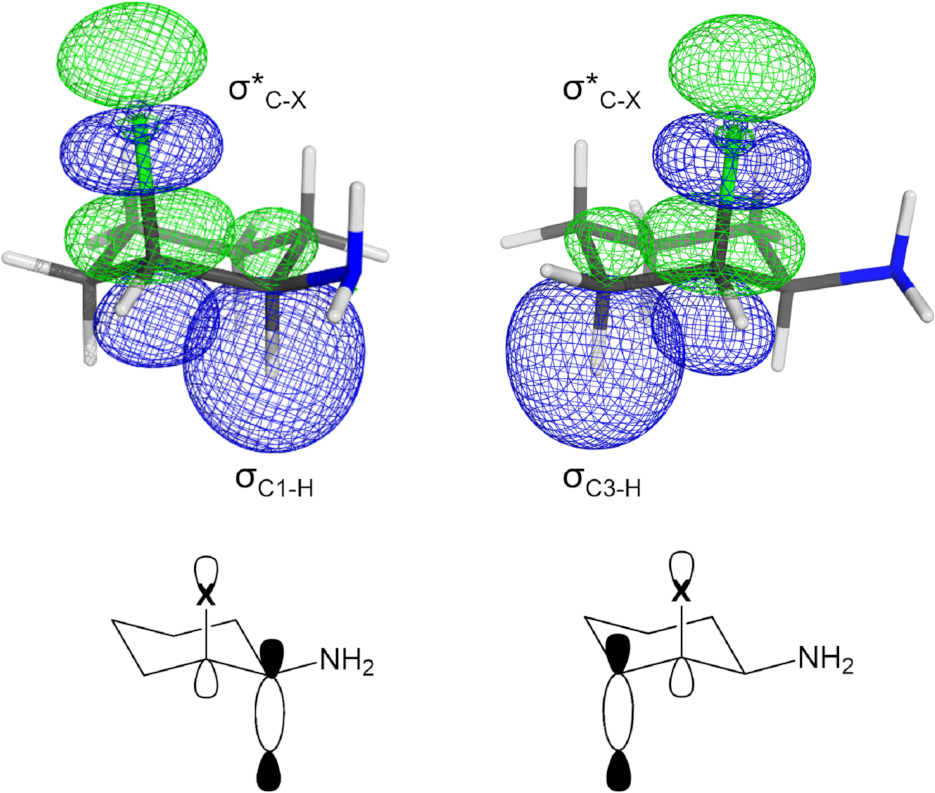
Orbitals overlap of σ_C1–H_ → σ*_C2–X_ (left) and σ_C3–Hax_ → σ*_C2–X_ (right) hyperconjugations in **ea** conformer.

As σ_C1–H_, σ*_C2–X_ and σ_C3–Hax_ are antiperiplanar, they favor orbital overlap, as illustrated in Figure 8. The orbital σ*_C–X_ acting as electron density acceptor of σ_C1–H_ and σ_C3–Hax_ can explain the great axial preference of the halogens. While in the **ea** conformer the donors σ_C1–H_ and σ_C3–Hax_ are antiperiplanar to the acceptor σ*_C–X_, in the **ae** conformer the corresponding donor orbitals are from C–C bonds (due to equatorial halogen orientation). Previous publications have already reported the C–H bond as slightly better donor than C–C bond [34–35].

Therefore, in the sum of interactions existent in the studied systems the hyperconjugation stands out, explaining the axial halogen and equatorial NH_2_ (**ea**) preference in all compounds.

## Conclusion

The conformational behavior of the *cis* isomers of 2-fluoro-, 2-chloro- and 2-bromocyclohexylamine was determined experimentally through DNMR at −80 °C, with populations of the **ea** conformer (equatorial amine group and axial halogen) higher than 90% in dichloromethane-*d*_2_ and methanol-*d*_4_. The conformational preference was affected neither by the solvent polarity nor the halogen size, suggesting the presence of strong stereoelectronic effects being responsible for the conformational behavior in these systems.

Theoretical calculations are in agreement with experimental data, the **ea** conformer being the most stable for all series, both in the gas phase and in solution. For the C–N rotation, the most unstable rotamers are *g*X for the **ae** conformer and *a* for **ea**, due to the orientation of the nitrogen lone pair electrons toward the halogen, which provides greater electrostatic repulsions.

The non-sensitivity to halogen size along the series can be attributed to a balance of steric, electrostatic and hyperconjugative interactions, being the strong conformational preference for **ea** explained by hyperconjugations involving mainly the C–X bond. The greater acceptor ability of the σ*_C–X_ orbital, axial in the **ea** conformer, enables strong interactions with the neighboring σ_C–H_ orbitals, and this acceptor ability increases along the halogen series (F < Cl < Br < I). However, interactions involving the halogen lone pair electrons follows the reverse order, counterbalancing the σ_C–H_ → σ*_C–X_ interactions along the series.

The strongly shifted and non-sensitive conformational equilibrium governed by a strong hyperconjugative interaction in *cis*-2-halocyclohexylamines shows that the conformational analysis of small molecules can still provide surprising results.

## Experimental

### Synthesis

*trans*-2-Fluorocyclohexanol was prepared according the literature [36] to provide *cis*-2-fluorocyclohexylamine.

*cis*-2-Fluorocyclohexylamine (**F**) was obtained by a Mitsunobu–Gabriel reaction, as described by Thvedt and co-workers [37]. Under nitrogen atmosphere, *trans*-2-fluorocyclohexanol (6.00 mmol), triphenylphosphine (6.60 mmol) and phthalimide (6.60 mmol) were dissolved in THF (40 mL). To this mixture was slowly added a solution of diisopropyl azodicarboxylate (DIAD, 40% in THF) and the mixture was stirred at room temperature for 18 h. The solvent was removed under reduced pressure and the reaction mixture was re-dissolved in CH_2_Cl_2_ (20 mL) and 10 mL of a 10% K_2_CO_3_ solution and stirred for 1 h. The resulting mixture was washed with distilled water (3 × 10 mL), the organic layer dried with Na_2_SO_4_ and concentrated under reduced pressure, and the crude product purified by silica-gel column chromatography (ethyl acetate/hexane 20%), resulting in 1.18 g (80%) of the *cis*-Gabriel amine. The free amine was obtained by the hydrazinolysis of the *cis*-Gabriel amine (0.40 mmol), in methanol (0.50 mL) with hydrazine hydrate (25%, 0.20 mL), stirred at room temperature for 24 h. To the mixture was added HCl until pH ≈ 2, forming a precipitate, which was filtered, and to the aqueous phase was added NaOH until pH ≈ 14 and the product extracted exhaustively (6 × 10 mL) with CH_2_Cl_2_. The organic layer was dried with Na_2_SO_4_ and the pure *cis*-2-fluorocyclohexylamine (0.19 g, 35%), a yellowish liquid concentrated under reduced pressure.

2-Chloro- and 2-bromocyclohexanone were synthetized as previously described [38–39], to provide *cis*-2-chloro (**Cl**) and *cis*-2-bromocyclohexylamine (**Br**), respectively, by a reductive amination [40].

To a sealed tube were added the corresponding ketone (10 mmol), ammonium acetate (100 mmol) and sodium cyanoborohydride (10 mmol) in methanol (30 mL) and the mixture stirred for 48 h at room temperature. To the mixture was added concentrated HCl until pH ≈ 2, forming a white precipitate (protonated amine), which was filtered and dissolved in distilled water, which was washed with CH_2_Cl_2_ (3 × 20 mL). To the aqueous layer was added NaOH until pH ≈ 14 and the product extracted exhaustively with CH_2_Cl_2_ (6 × 20 mL). The crude mixture was purified by silica-gel column chromatography (acetone/chloroform 10% for **Cl** and 25% for **Br**), and the *cis* isomer was isolated corresponding to a yellowish liquid for **Cl** (0.04 g, 15%) and a white amorphous solid for **Br** (0.03 g, 10%).

***cis*-2-Fluorocyclohexylamine:** ^1^H NMR (500.13 MHz, methanol-*d*_4_) δ 4.64 (dddd, 1H, H_2_), 2.70 (dddd, 1H, H_1_), 2.01 (m, 1H, 1H_3_), 1.74–1.64 (m, 2H, 1H_5_, 1H_6_), 1.61–1.42 (m, 4H, 1H_3_, 2H_4_, 1H_6_), 1.34 (m, 1H, H_5_); ^13^C NMR (125.77 MHz, methanol-*d*_4_) δ 93.58 (C_2_), 52.80 (C_1_), 30.84 (C_3_, C_6_), 20.85 (C_4_), 24.88 (C_5_); HRMS (ESI/QTOF) *m*/*z*: [M + H]^+^ calcd for C_6_H_12_FN, 118.0953; found, 118.0991.

***cis*-2-Chlorocyclohexylamine: **^1^H NMR (500.13 MHz, dichloromethane-*d*_2_) δ 4.30 (ddd, 1H, H_2_), 2.80 (ddd, 1H, H_1_), 2.04 (m, 1H, 1H_3_), 1.78 (m, 1H, 1H_3_), 1.70–1.60 (m, 2H, 1H_5_, 1H_4_), 1,54 (m, 2H, 2H_6_), 1.40 (m, 1H, 1H_4_), 1.30 (m, 1H, 1H_5_); ^13^C NMR (125.77 MHz, dichlorotmethane-*d*_2_) δ 68.47 (C_2_), 53.22 (C_1_), 33.16 (C_3_), 31.30 (C_6_), 23.76 (C_5_), 21.10 (C_4_); HRMS (ESI/QTOF) *m*/*z*: [M + H]^+^ calcd for C_6_H_12_ClN, 134.0658; found, 134.0692.

***cis*-2-Bromocyclohexylamine: **^1^H NMR (500.13 MHz, dichloromethane-*d*_2_) δ 4.68 (ddd, 1H, H_2_), 2.97 (ddd, 1H, H_1_), 2.18 (m, 1H, H_3_), 1.93 (m, 1H, H_3_), 1.77–1.63 (m, 4H, 1H_4_, 1H_5_, 2H_6_), 1.50 (m, 1H, H_4_), 1.37 (m, 1H, H5); ^13^C NMR (125.77 MHz, dichlorotmethane-*d*_2_) δ 62.19 (C_1_), 53.42 (C_2_), 33.67 (C_3_), 30.08 (C_5_), 23.87 (C_6_), 21.28 (C_4_); HRMS (ESI/QTOF) *m*/*z*: [M + H]^+^ calcd for C_6_H_12_BrN, 178.0153; found, 178.0189.

### NMR experiments

¹H and ¹³C NMR spectra were acquired on a Bruker Avance III HD spectrometer, operating at 500.13 MHz for ^1^H nuclei and 125.77 MHz for ^13^C nuclei in solutions with approximate concentration of 0.01 mol L^–1^ in dichloromethane-*d*_2_ and methanol-*d*_4_, using tetramethylsilane as internal reference. The probe was coupled to a liquid nitrogen evaporator system to decrease the temperature from 25 to −80 °C. Typical ^1^H NMR spectra were run with a spectral window of approximately 10000 Hz (20 ppm) for ^1^H spectra, and 30000 Hz (238 ppm) for ^13^C spectra, with number of points 32k, resulting in a digital resolution of 0.12 Hz/point. The spectral data are available in Supporting Information File 1.

### Theoretical calculations

Theoretical calculations were performed with the software package Gaussian 09 [41]. To determine the lowest energy structures for each conformer, we built a PES (in M06-2X/aug-cc-pVDZ), varying the C2–C1–N–H dihedral angle in steps of 15° manually, to avoid nitrogen inversion.

Optimization and frequency calculations were performed for the three lower-energy rotamers for each conformer, both in the gas phase and in solution (Cartesian coordinates are available in Supporting Information File 1). The calculations were performed using the M06-2X density functional method, and the second-order Möller–Plesset perturbation method (MP2), associated with the Pople 6-311++G(2df,2p), and Dunning aug-cc-pVDZ basis set. The mixed basis set function LanL2DZ-ECP was employed for the iodine atom. Solvation calculations were performed in dichloromethane and methanol, with the the implicit model IEF-PCM and the description of the molecular cavity through Bondi’s atomic radii.

Molecular Mechanics calculations were performed using the software Avogadro 1.2.0 [42], employing the Amber force field GAFF [33].

NBO calculations were performed in the optimized structures in the gas phase (M06-2X/6-311++G(2df,2p)), through the module NBO 5.9 [43] from Gaussian 09. For the PCA, it was used the software R, version 3.5.0 [44], with the graphical interface RStudio, version 1.0.153 [45].

## Supporting Information

File 1 ^1^H and ^13^C NMR spectra at 25 and −80 °C, supplementary theoretical values, RScript for PCA and the Cartesian coordinates of the structures.

File 2 NBO data for PCA.
