# Supplementary material for: Strong hyperconjugative interactions limit solvent and substituent influence on conformational equilibrium: the case of cis-2-halocyclohexylamines
Source: Beilstein J Org Chem. 2019 Apr 1;15:818–29. doi: 10.3762/bjoc.15.79 (PMC6466766; doi:10.3762/bjoc.15.79)
Supplement: File 1 — 1H and 13C NMR spectra at 25 and −80 °C, supplementary theoretical values, RScript for PCA and the Cartesian coordinates of the structures. [file Beilstein_J_Org_Chem-15-818-s001.pdf]

BEILSTEIN JOURNAL OF ORGANIC CHEMISTRY

Supporting Information

for

Strong hyperconjugative interactions limit solvent and substituent influence on conformational equilibrium: the case of *cis*-2-halocyclohexylamines

Camila B. Francisco, Cleverton S. Fernandes, Ulisses Z. de Melo, Roberto Rittner, Gisele F. Gauze and Ernani A. Basso

Beilstein J. Org. Chem. **2019,** *15,* 818–829. doi:10.3762/bjoc.15.79

^{1}H and ^{13}C NMR spectra at 25 and −80 °C, supplementary theoretical values, RScript for PCA and the Cartesian coordinates of the structures

License and Terms: This is a supporting information file under the terms of the Creative Commons Attribution License (http://creativecommons.org/licenses/by/4.0). Please note that the reuse, redistribution and reproduction in particular requires that the authors and source are credited.
The license is subject to the *Beilstein Journal of Organic Chemistry* terms and conditions: (https://www.beilstein-journals.org/bjoc)

Table of Contents

A. NMR Data (^{1}H and ^{13}C)

Figure A1. ^{1}H NMR spectra (500.13 MHz) of *trans*-2-fluorocyclohexanol in chloroform-*d* at 25 °C S1
Figure A2. ^{1}H NMR spectra (500.13 MHz) of 2-chlorocyclohexanone in chloroform-*d* at 25 °C S2
Figure A3. ^{1}H NMR spectra (500.13 MHz) of 2-bromocyclohexanone in chloroform-*d* at 25 °C S3
Figure A4. ^{1}H NMR spectra (500.13 MHz) of *cis*-2-fluorocyclohexylamine in methanol-d_4 at 25 °C S4
Figure A5. ^{13}C NMR spectra (125.77 MHz) of *cis*-2-fluorocyclohexylamine in methanol-d_4 at 25 °C S5
Figure A6. ^{1}H NMR spectra (500.13 MHz) of *cis*-2-fluorocyclohexylamine in dichloromethane-d_2 at −80 °C S6
Figure A7. ^{13}C NMR spectra (125.77 MHz) of *cis*-2-fluorocyclohexylamine in dichloromethane-d_2 at −80 °C S7
Figure A8. ^{1}H NMR spectra (500.13 MHz) of *cis*-2-fluorocyclohexylamine in methanol-d_4 at −80 °C S8
Figure A9. ^{13}C NMR spectra (125.77 MHz) of *cis*-2-fluorocyclohexylamine in methanol-d_4 at −80 °C S9
Figure A10. ^{1}H NMR spectra (500.13 MHz) of *cis*-2-chlorocyclohexylamine in dichloromethane-d_2 at 25 °C S10
Figure A11. ^{13}C NMR spectra (125.77 MHz) of *cis*-2-chlorocyclohexylamine in dichloromethane-d_2 at 25 °C S11
Figure A12. ^{1}H NMR spectra (500.13 MHz) of *cis*-2-chlorocyclohexylamine in dichloromethane-d_2 at −80 °C S12
Figure A13. ^{13}C NMR spectra (125.77 MHz) of *cis*-2-chlorocyclohexylamine in dichloromethane-d_2 at −80 °C S13
Figure A14. ^{1}H NMR spectra (500.13 MHz) of *cis*-2-chlorocyclohexylamine in methanol-d_4 at −80 °C S14
Figure A15. ^{13}C NMR spectra (125.77 MHz) of *cis*-2-chlorocyclohexylamine in methanol-d_4 at −80 °C S15
Figura A16. ^{1}H NMR spectra (500.13 MHz) of *cis*-2-bromocyclohexylamine in dichloromethane-d_2 at 25 °C S16
Figure A17. ^{13}C NMR spectra (125.77 MHz) of *cis*-2-bromocyclohexylamine in dichloromethane-d_2 at 25 °C S17
Figure A18. ^{1}H NMR spectra (500.13 MHz) of *cis*-2-bromocyclohexylamine in dichloromethane-d_2 at -80 °C S18
Figure A19. ^{13}C NMR spectra (125.77 MHz) of *cis*-2-bromocyclohexylamine in dichloromethane-d_2 at -80 °C S19

Figure A20. ^{1}H NMR spectra (500.13 MHz) of *cis*-2-bromocyclohexylamine in methanol-d_4 at −80 °C S20
Figure A21. ^{13}C NMR spectra (125.77 MHz) of *cis*-2-bromocyclohexylamine in methanol-d_4 at −80 °C S21

B. Theoretical calculations data

Table B1. Δ*E* values (kcal mol^{-1}) for all rotamers of *cis*-2-halocyclohexylamines in different theory levels in gas phase and in dichloromethane and methanol……………………………………………………………………….S22

Table B2. Δ*G*º values for **ea** conformer of *cis*-2-halocyclohexylamines calculated in different theory levels in gas phase and in dichloromethane and methanol………………………………………………………………………S23

Table B3. Variation in total energy (ΔE_{total}), localized energy (ΔE_{loc}), delocalization energy (ΔE_{deloc}) steric exchange energy (ΔE_{steric}) and in electrostatic energy (ΔE_{elect}) in kcal mol^{-1}, for all rotamers in **ae** and **ea** conformers of *cis*-2-halocyclohexylamines calculated in gas phase (M06-2X/6-311++G(2df,2p)……………………………………………………………… S23

C. Principal component analysis data ………………………………………..…S24

D. Cartesian coordinates…………………………………………….……….S25

A. NMR Data

Figure A1. ^{1}H NMR spectra (500.13 MHz) of *trans*-2-fluorocyclohexanol in chloroform-*d* at 25 °C

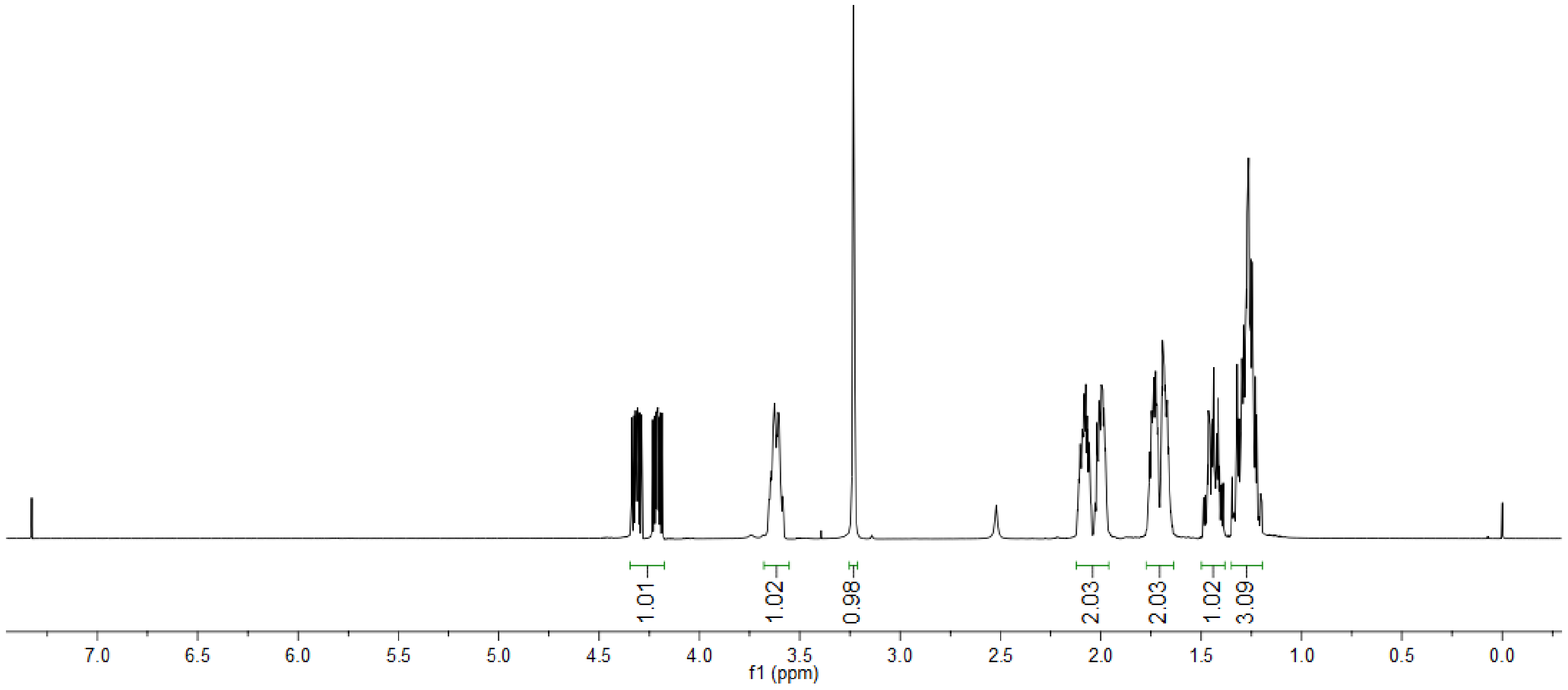

Figure A2. ^{1}H NMR spectra (500.13 MHz) of 2-chlorocyclohexanone in chloroform-*d* at 25 °C

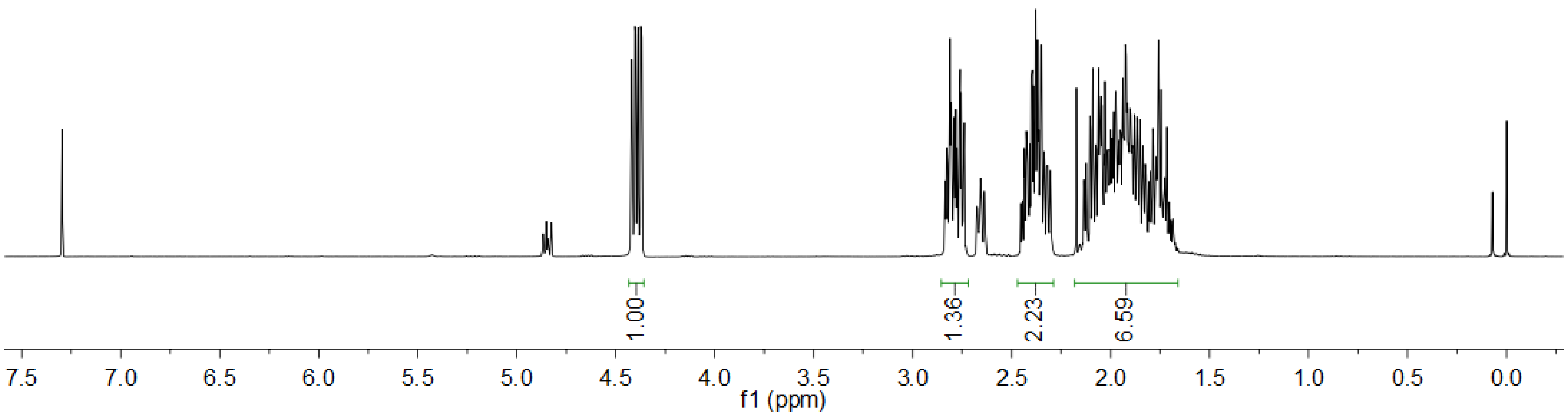

Figure A3. ^{1}H NMR spectra (500.13 MHz) of 2-bromocyclohexanone in chloroform-*d* at 25 °C

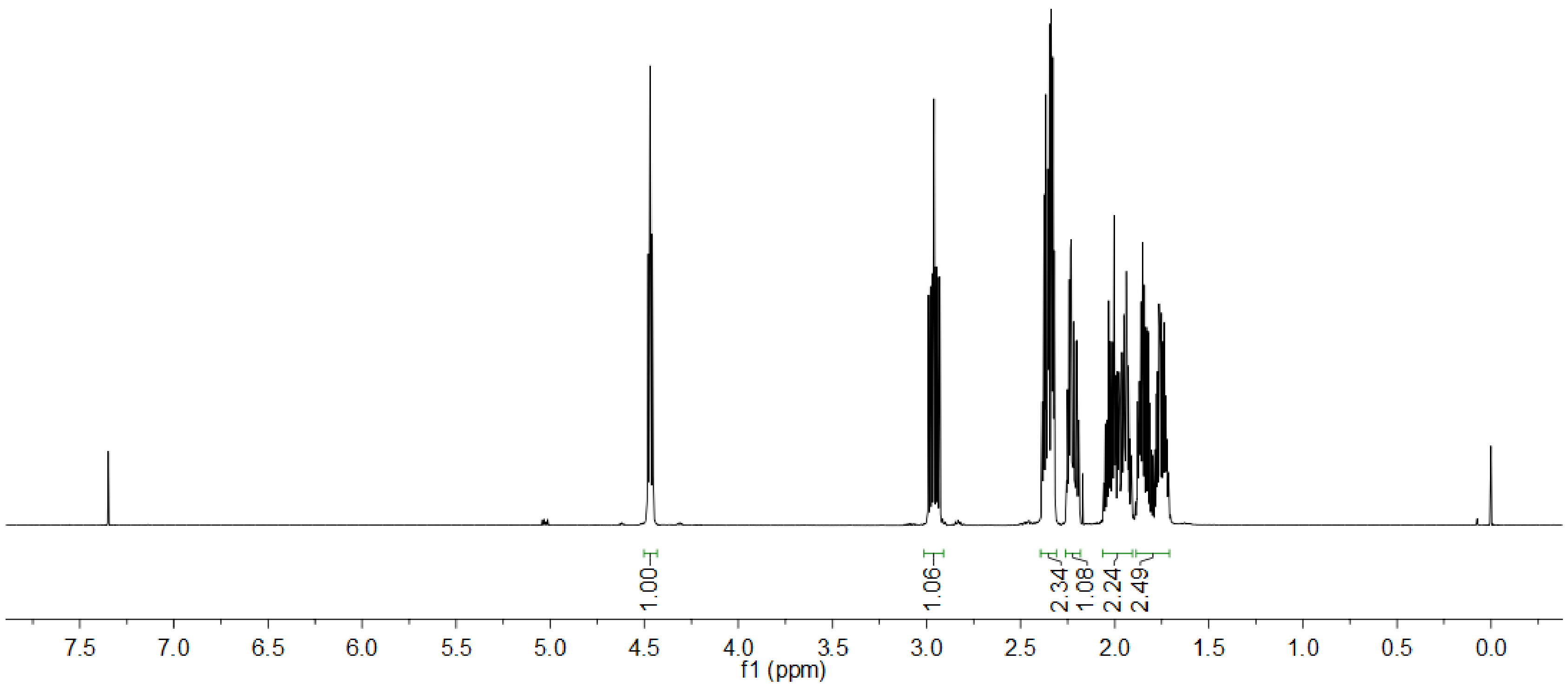

Figure A4. ^{1}H NMR spectra (500.13 MHz) of *cis*-2-fluorocyclohexylamine in methanol-d_4 at 25 °C

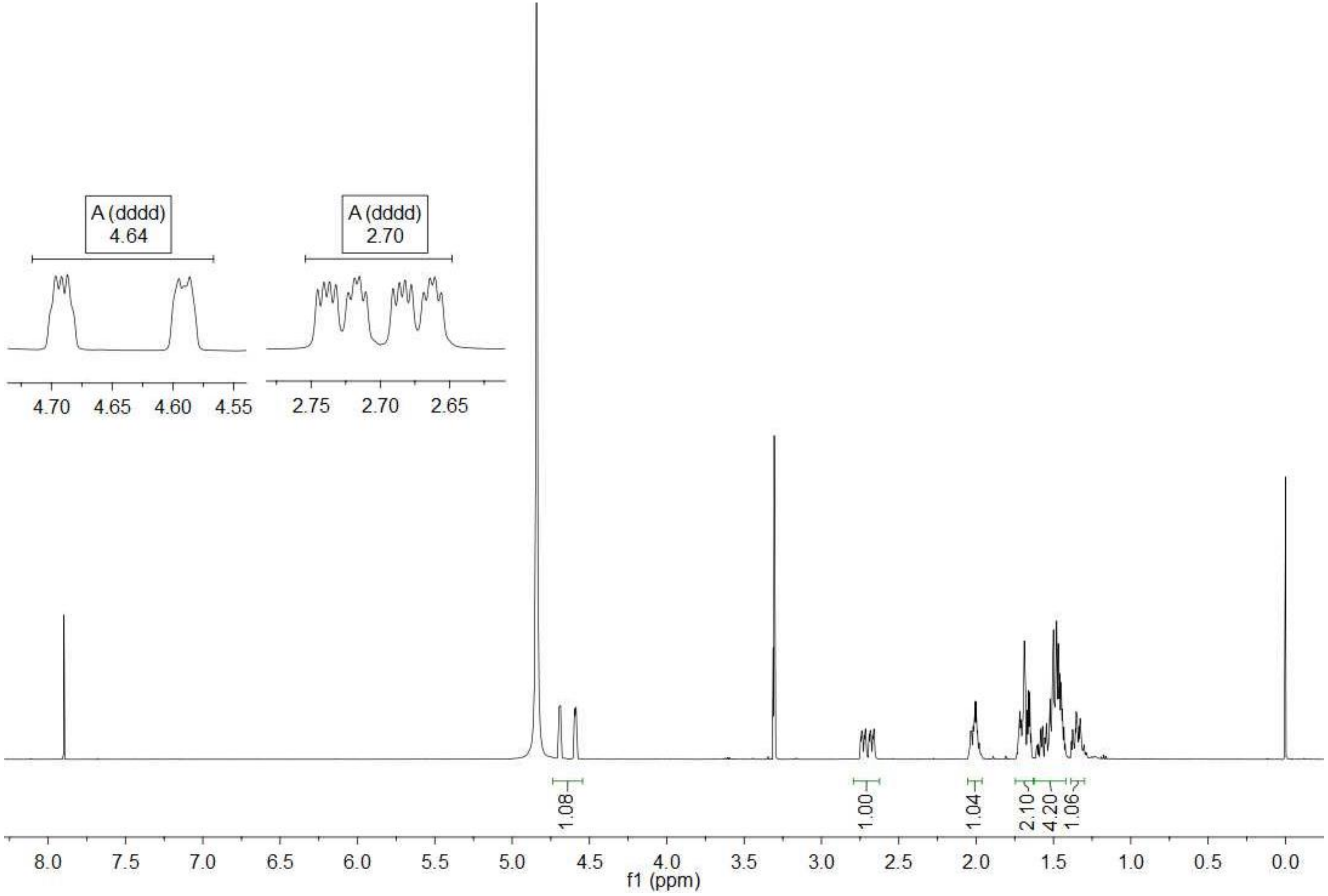

Figure A5. ^{13}C NMR spectra (125.77 MHz) of *cis*-2-fluorocyclohexylamine in methanol-d_4 at 25 °C

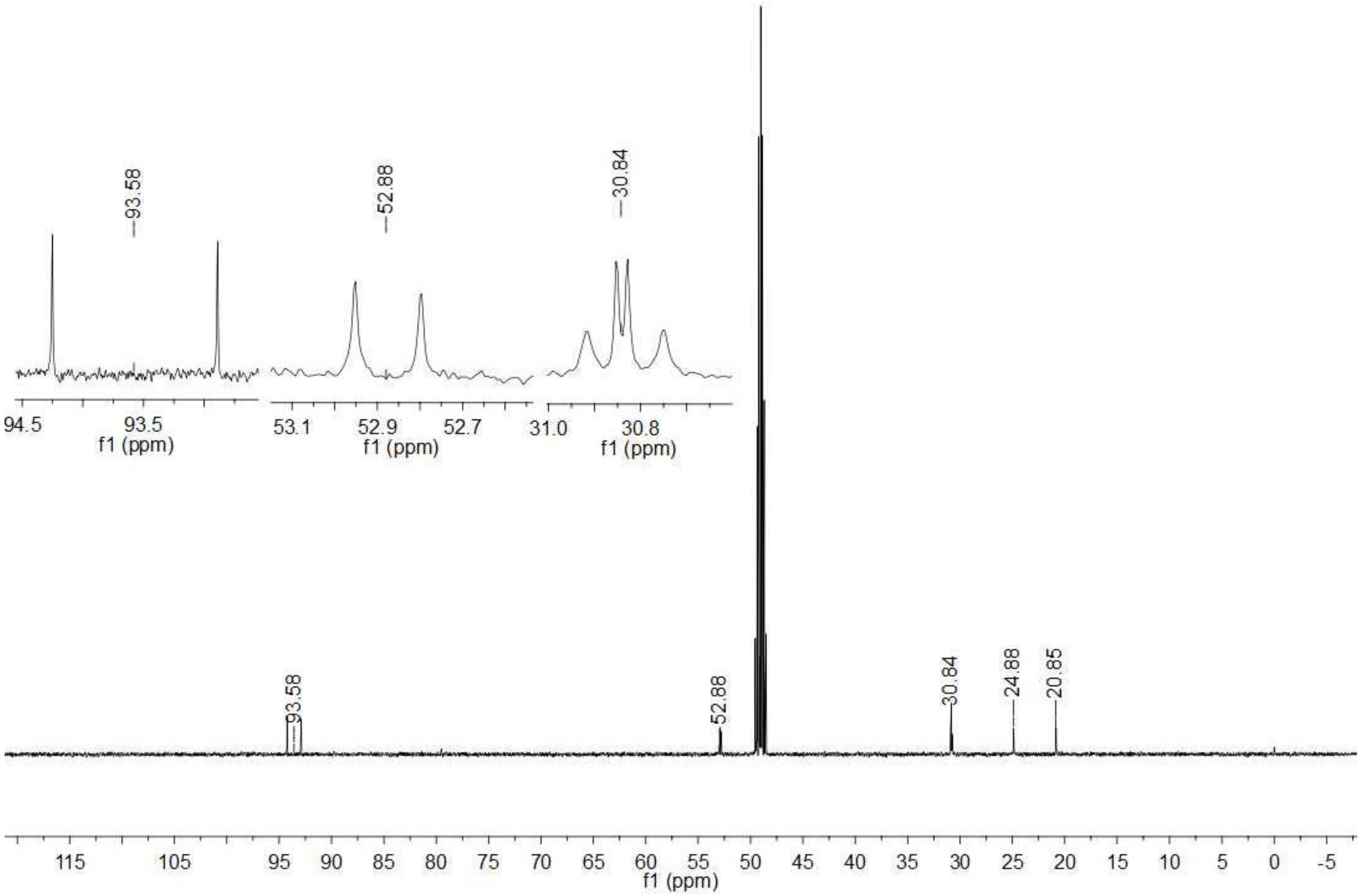

Figure A6. ^{1}H NMR spectra (500.13 MHz) of *cis*-2-fluorocyclohexylamine in dichloromethane-d_2 at −80 °C

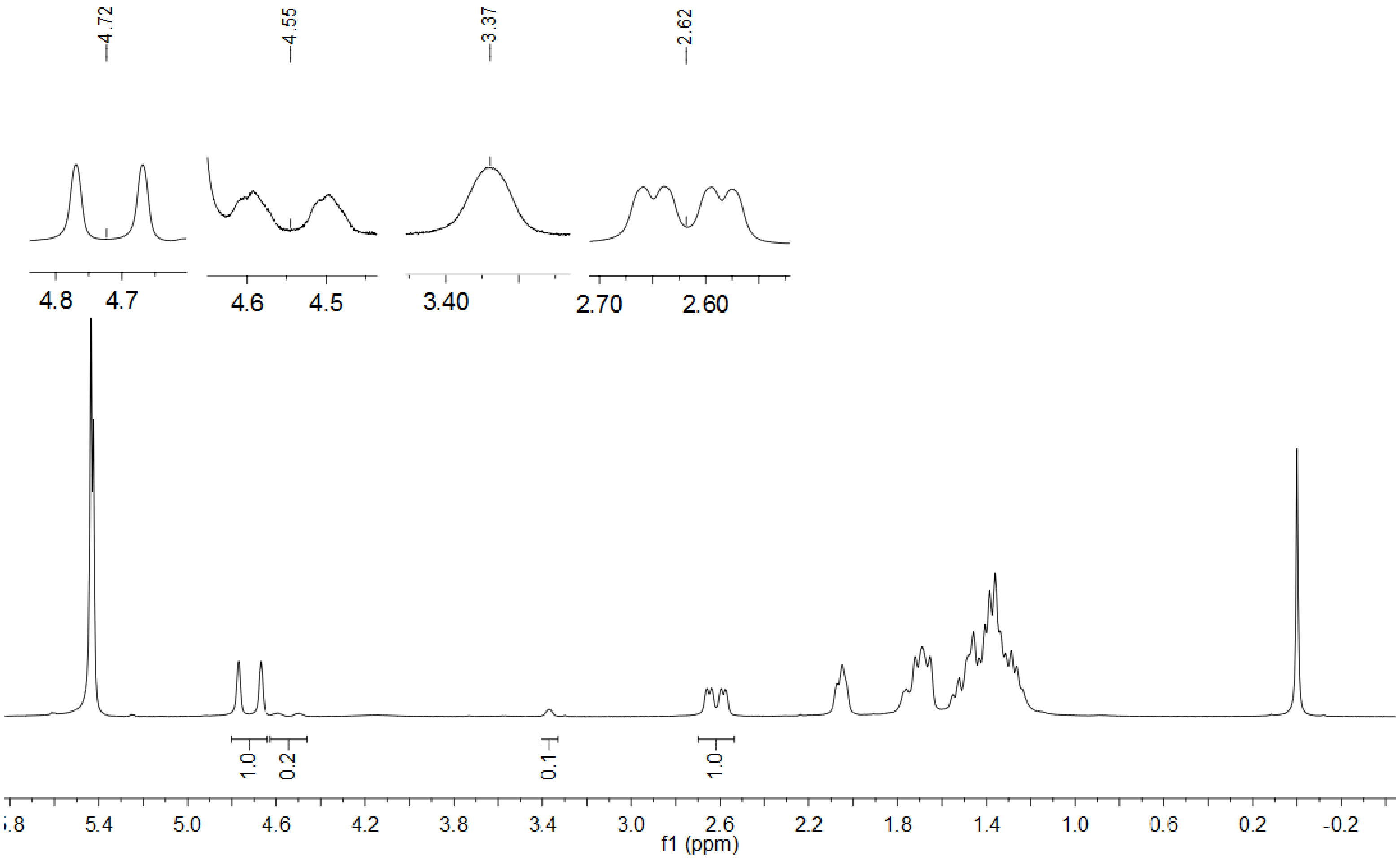

Figure A7. ^{13}C NMR spectra (125.77 MHz) of *cis*-2-fluorocyclohexylamine in dichloromethane-d_2 at −80 °C

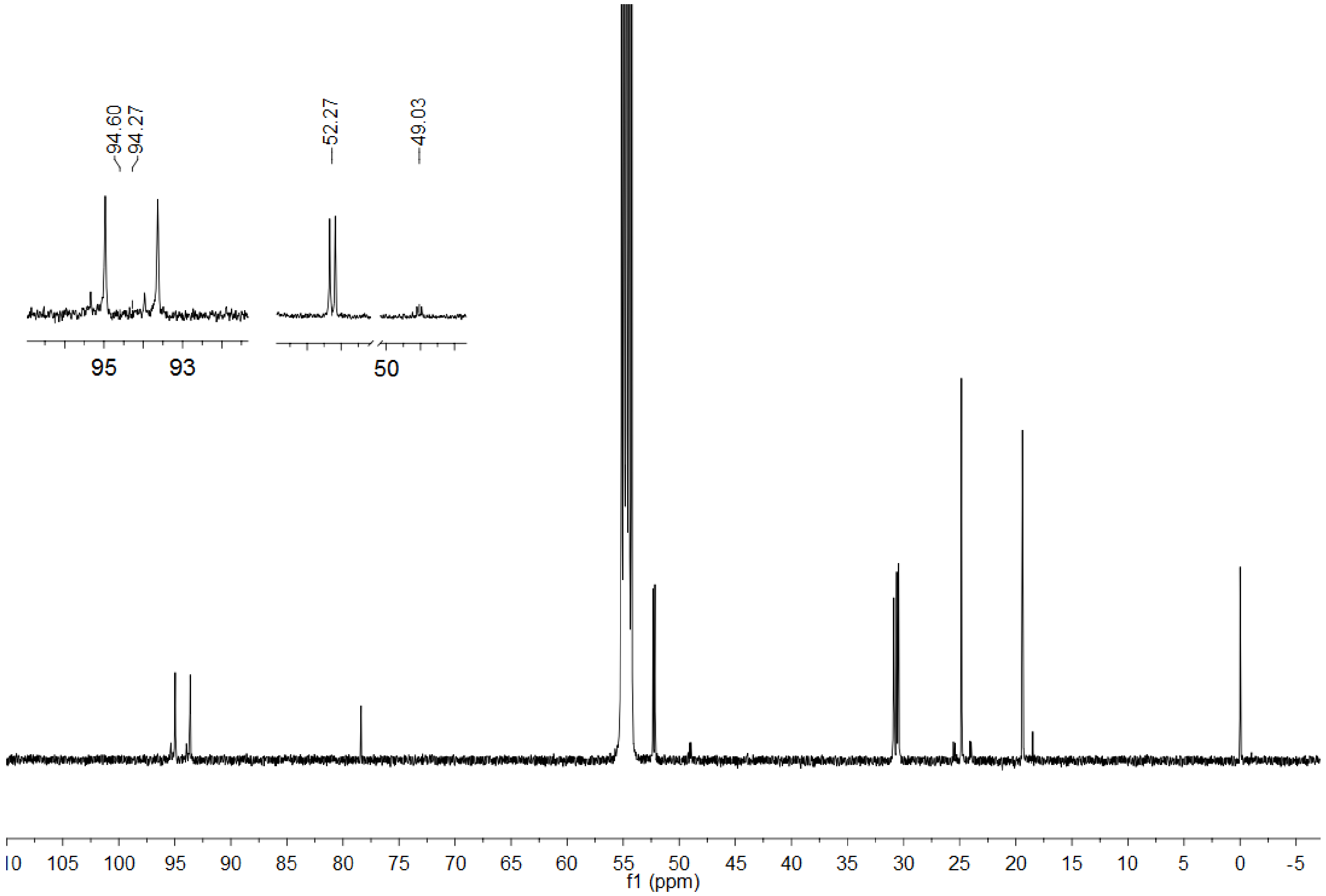

Figure A8. ^{1}H NMR spectra (500.13 MHz) of *cis*-2-fluorocyclohexylamine in methanol-d_4 at −80 °C

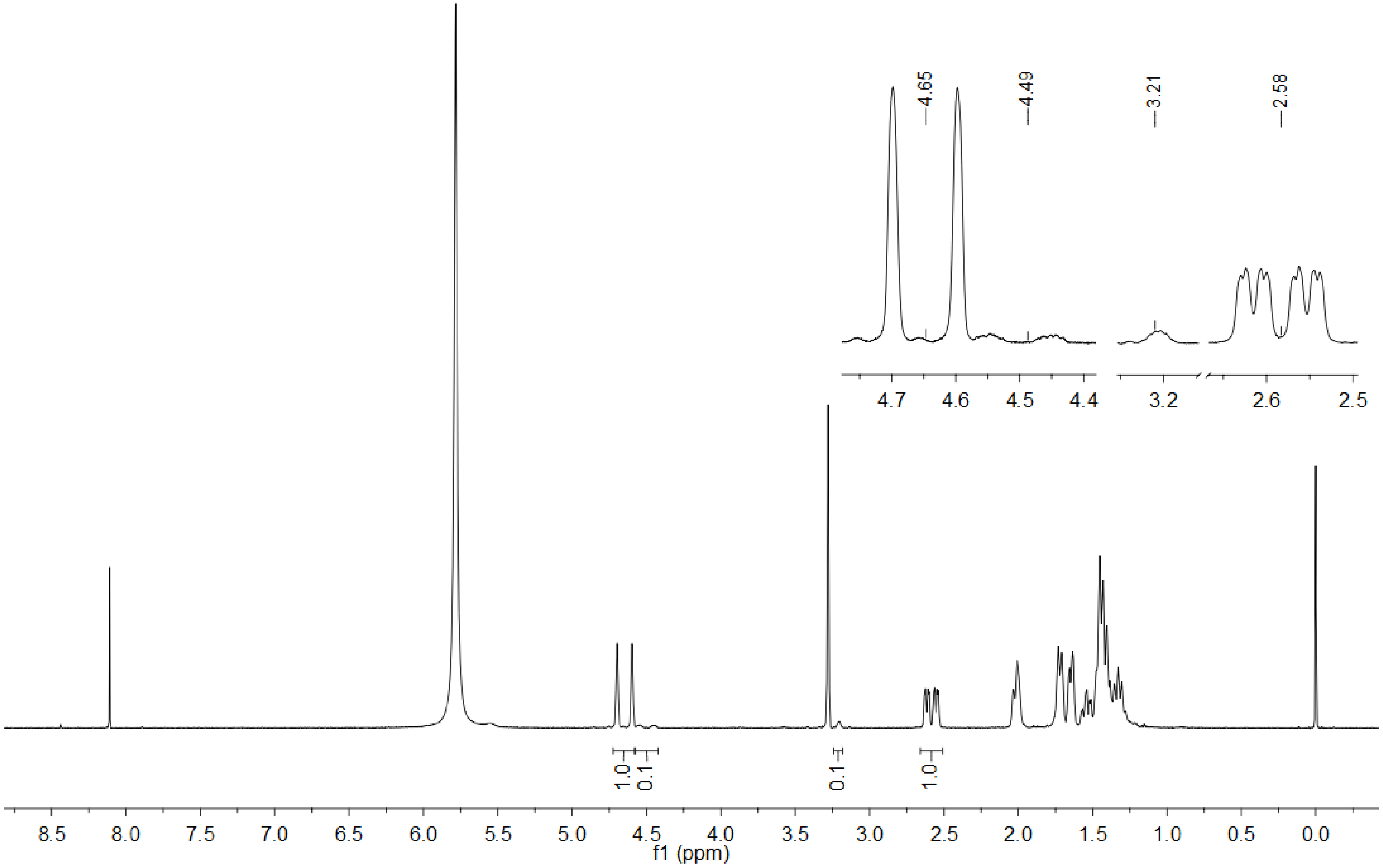

Figure A9. ^{13}C NMR spectra (125.77 MHz) of *cis*-2-fluorocyclohexylamine in methanol-d_4 at −80 °C

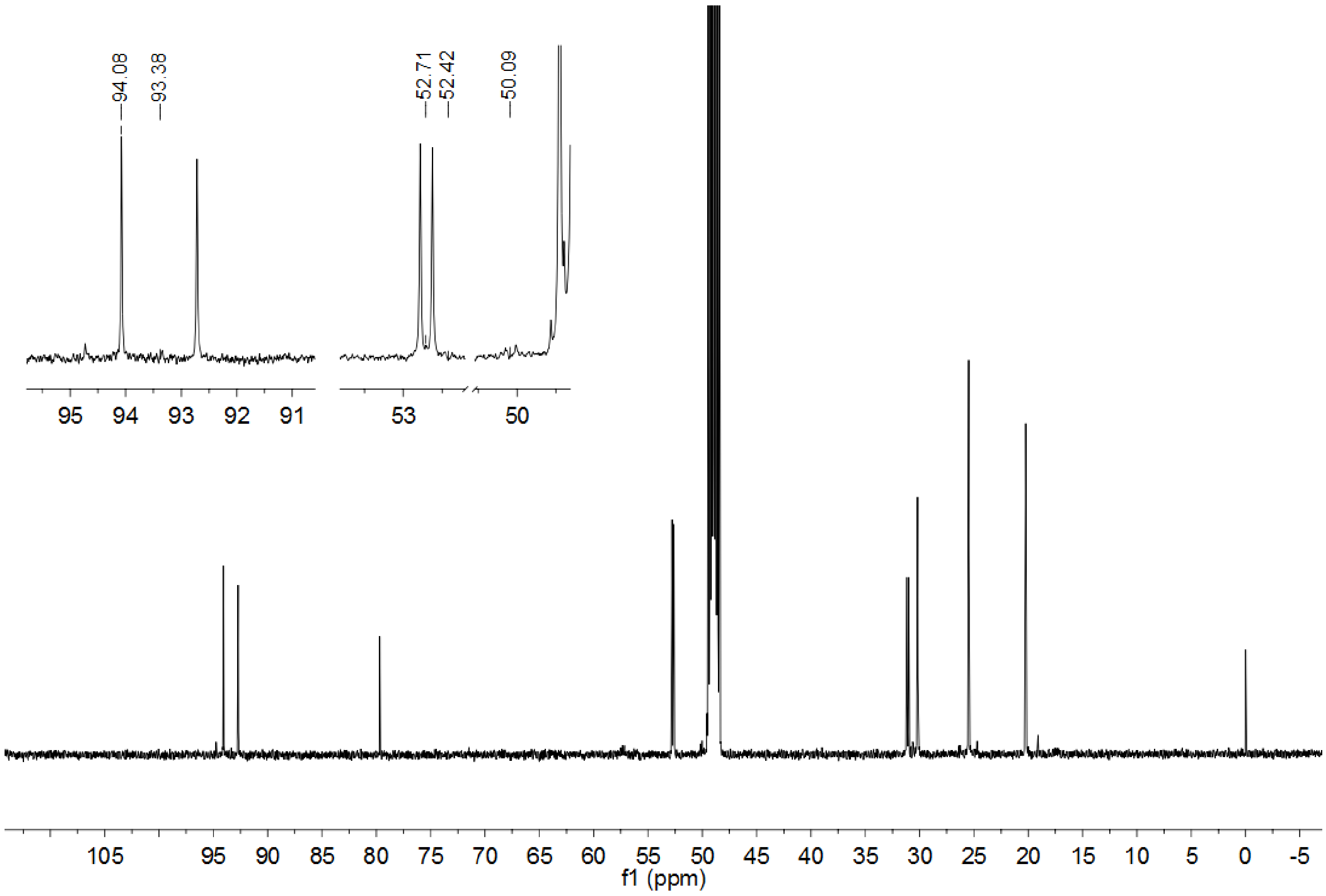

Figure A10. ^{1}H NMR spectra (500.13 MHz) of *cis*-2-chlorocyclohexylamine in dichloromethane-d_2 at 25 °C

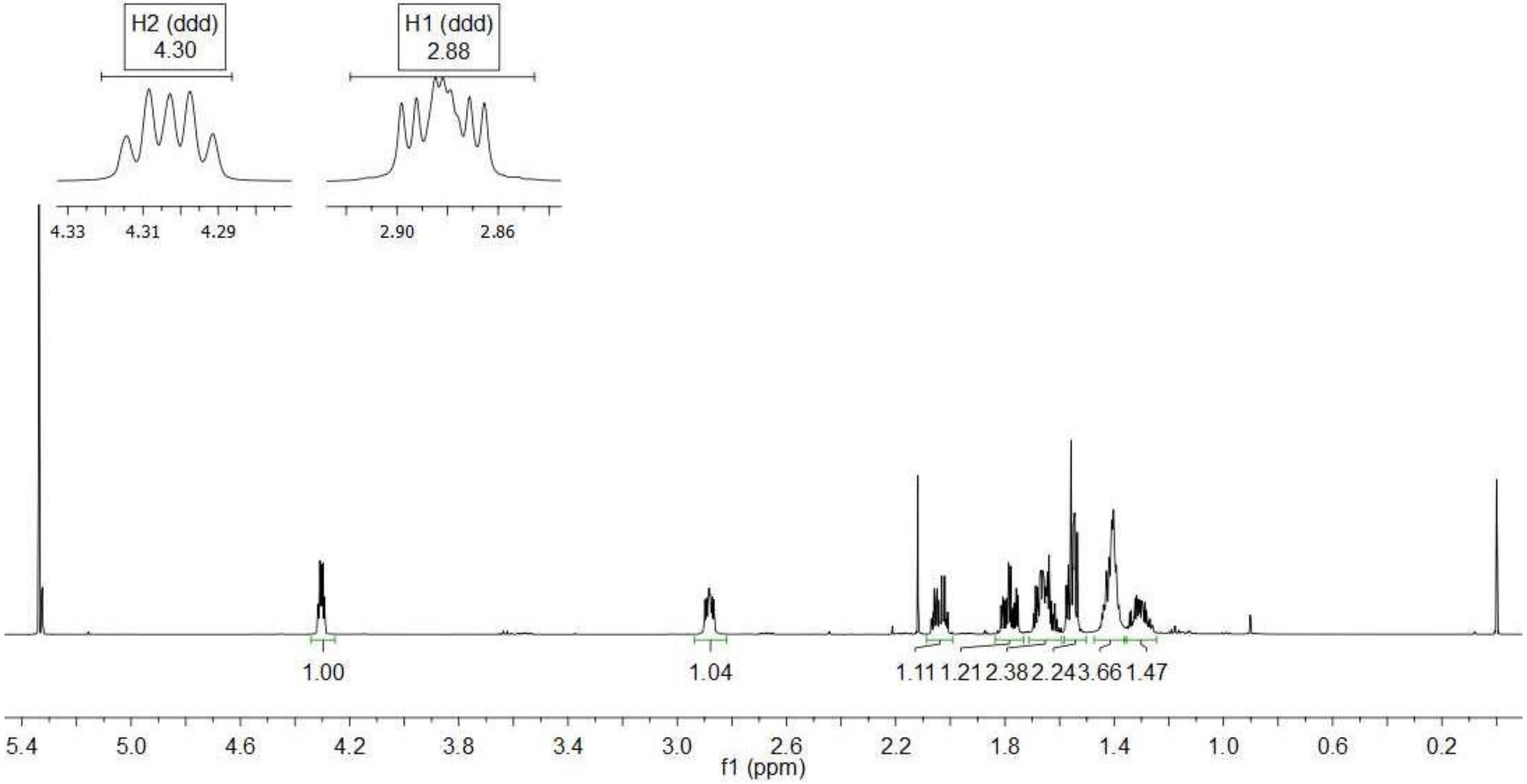

Figure A11. ^{13}C NMR spectra (125.77 MHz) of *cis*-2-chlorocyclohexylamine in dichloromethane-d_2 at 25 °C

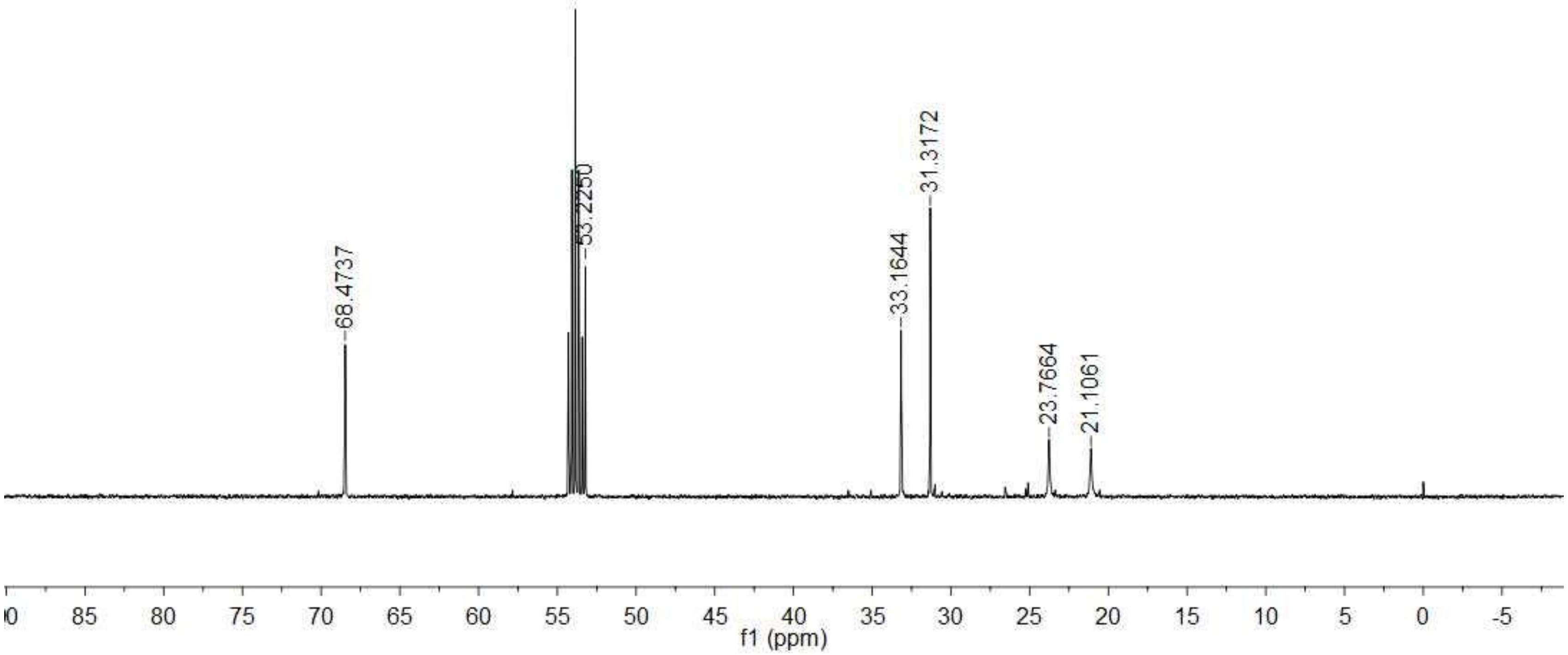

Figure A12. ^{1}H NMR spectra (500.13 MHz) of *cis*-2-chlorocyclohexylamine in dichloromethane-d_2 at −80 °C

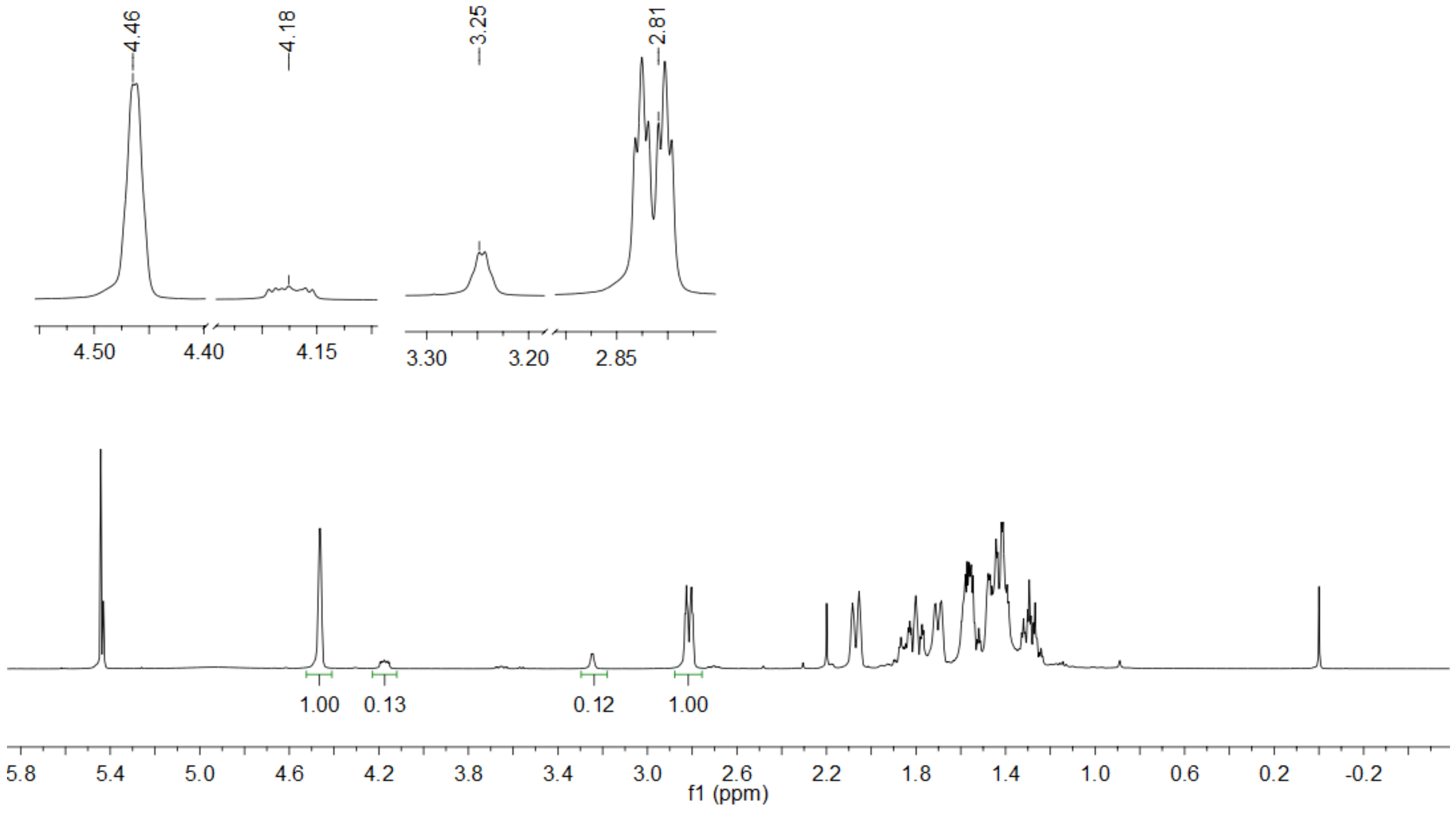

Figure A13. ^{13}C NMR spectra (125.77 MHz) of *cis*-2-chlorocyclohexylamine in dichloromethane-d_2 at −80 °C

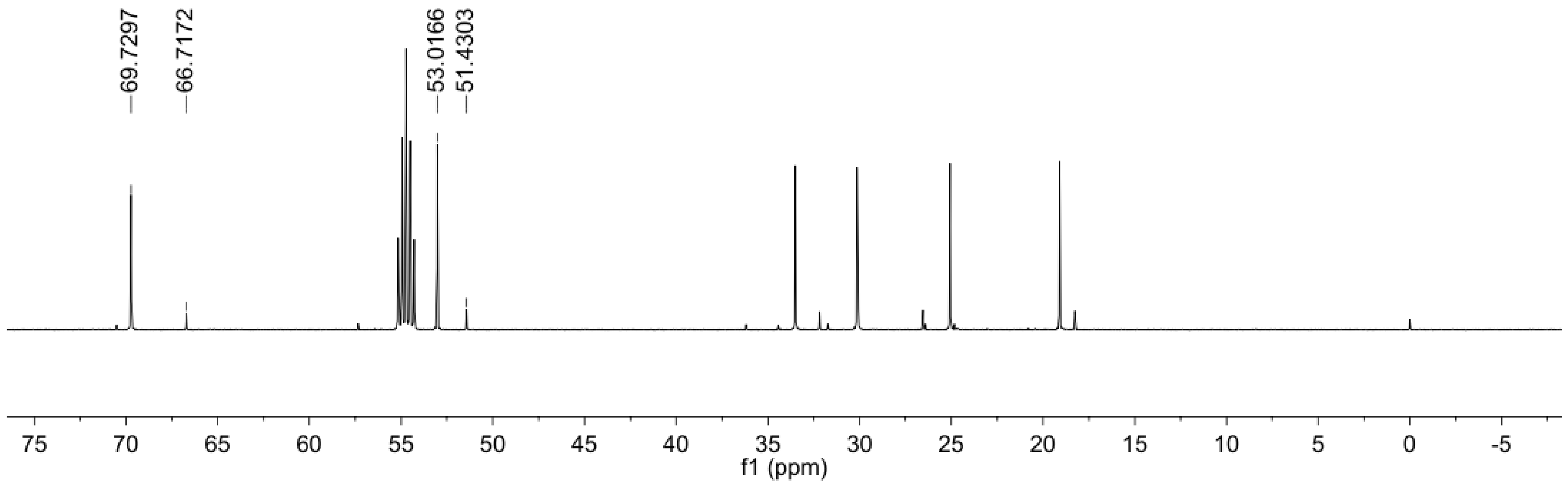

Figure A14. ^{1}H NMR spectra (500.13 MHz) of *cis*-2-chlorocyclohexylamine in methanol-d_4 at −80 °C

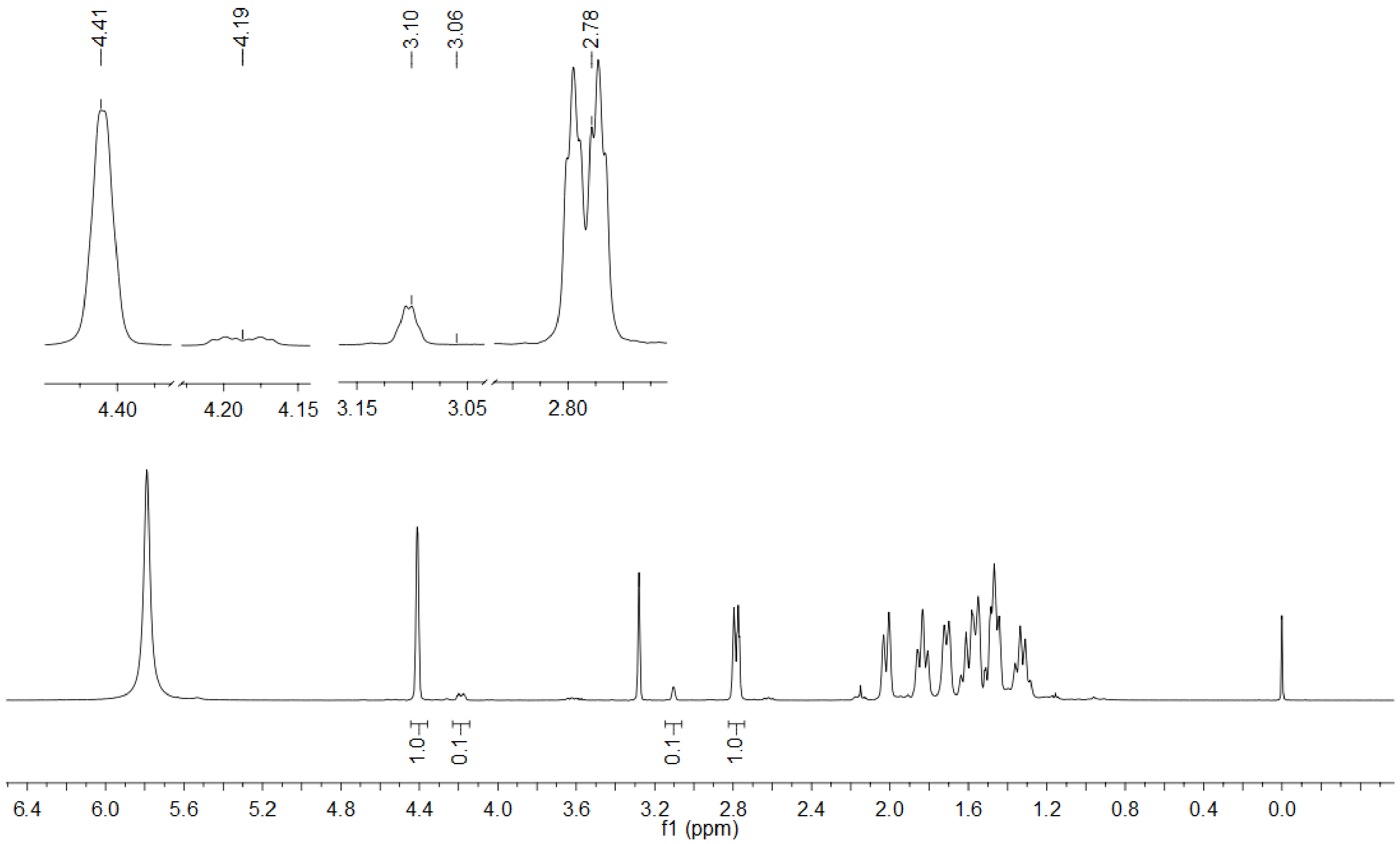

Figure A15. ^{13}C NMR spectra (125.77 MHz) of *cis*-2-chlorocyclohexylamine in methanol-d_4 at −80 °C

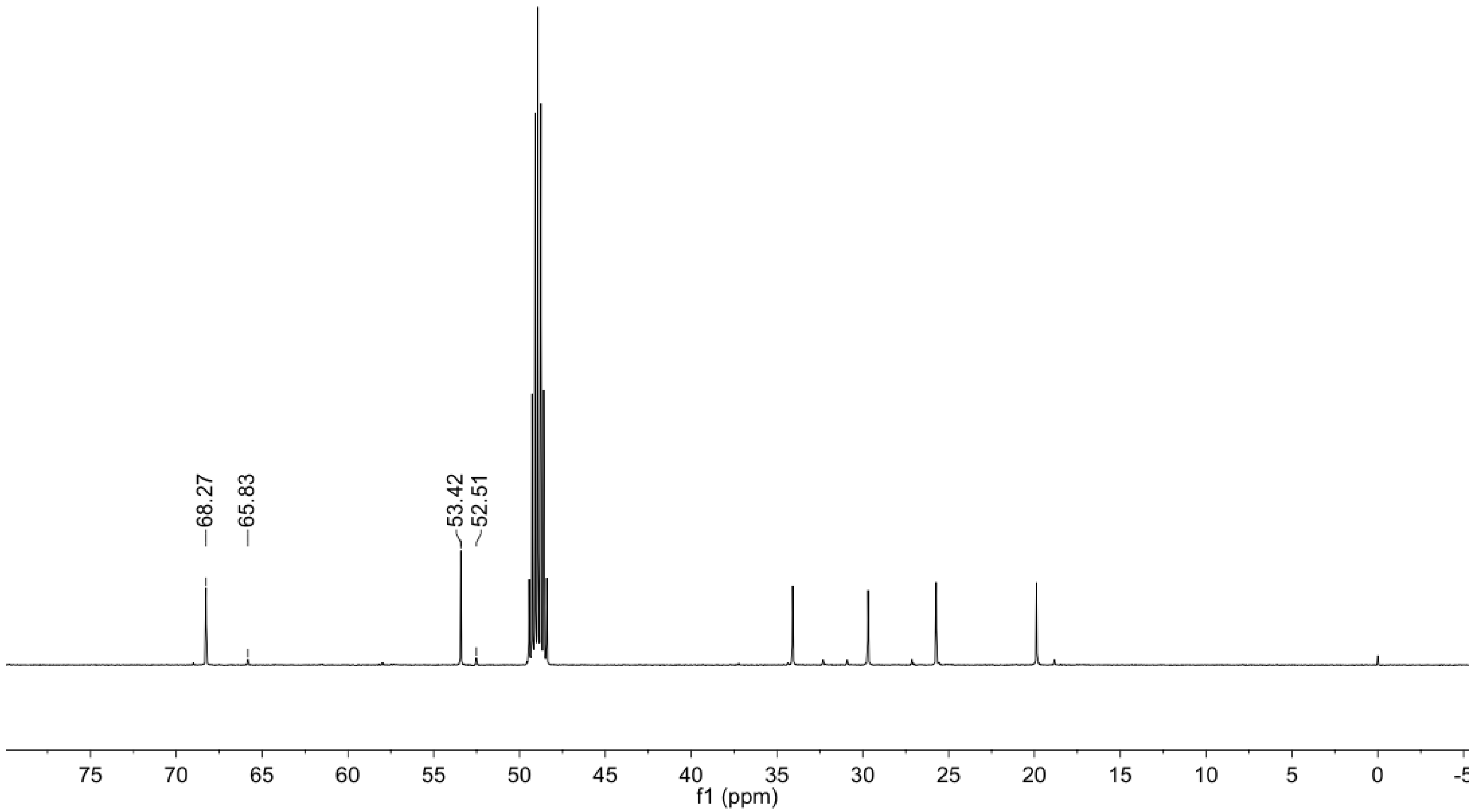

Figura A16. ^{1}H NMR spectra (500.13 MHz) of *cis*-2-bromocyclohexylamine in dichloromethane-d_2 at 25 °C

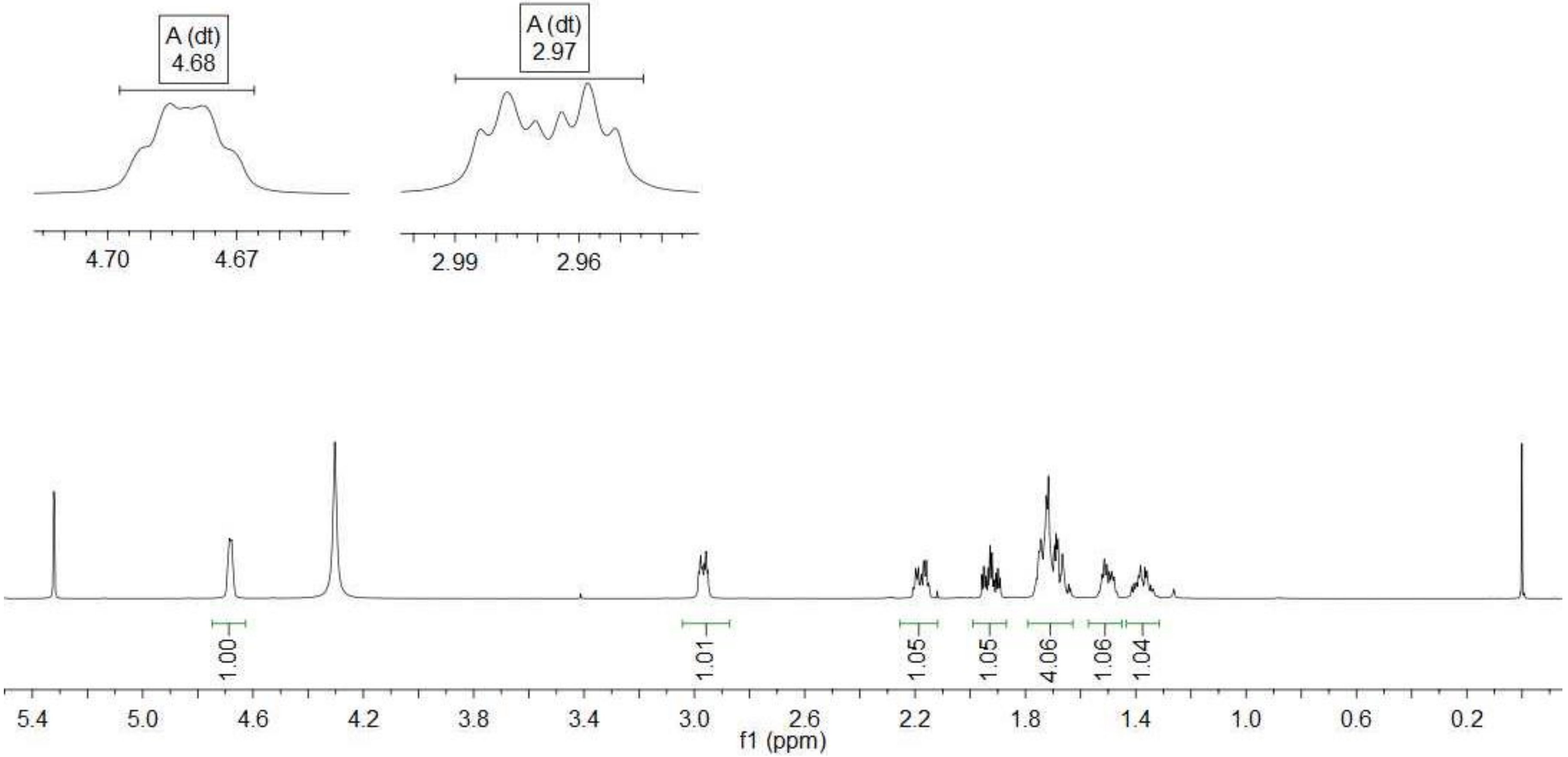

Figure A17. ^{13}C NMR spectra (125.77 MHz) of *cis*-2-bromocyclohexylamine in dichloromethane-d_2 at 25 °C

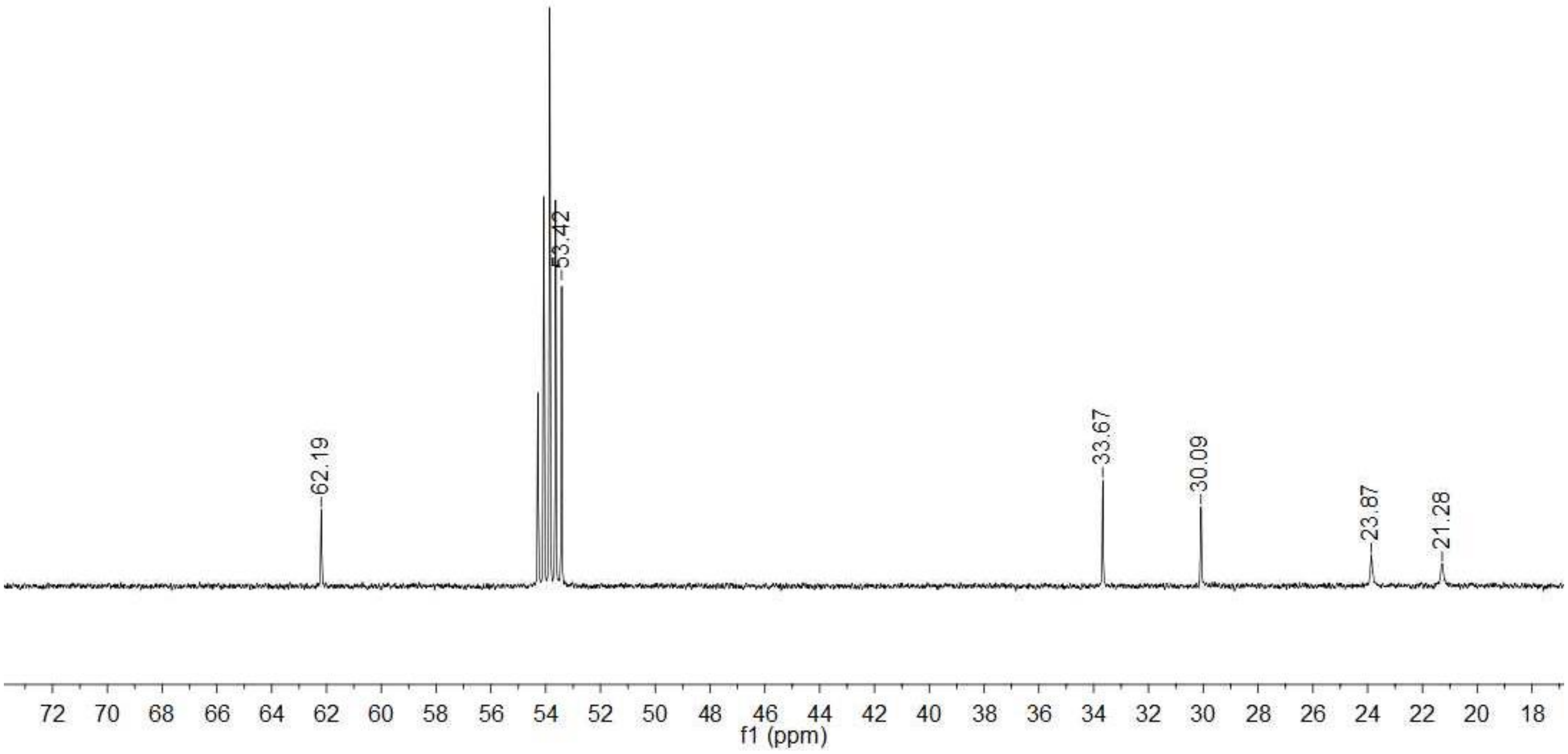

Figure A18. ^{1}H NMR spectra (500.13 MHz) of *cis*-2-bromocyclohexylamine in dichloromethane-d_2 at −80 °C

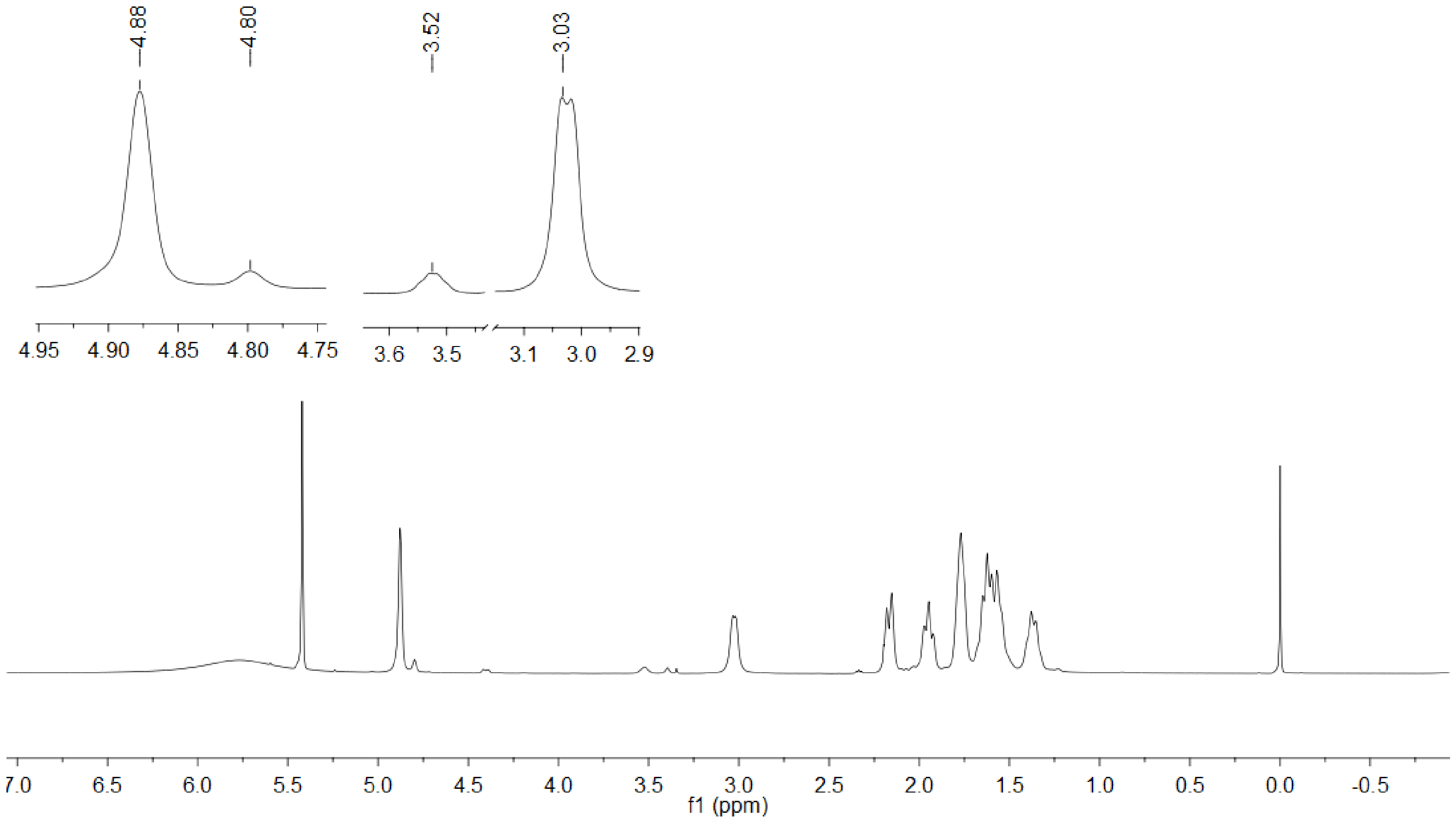

Figure A19. ^{13}C NMR spectra (125.77 MHz) of *cis*-2-bromocyclohexylamine in dichloromethane-d_2 at −80 °C

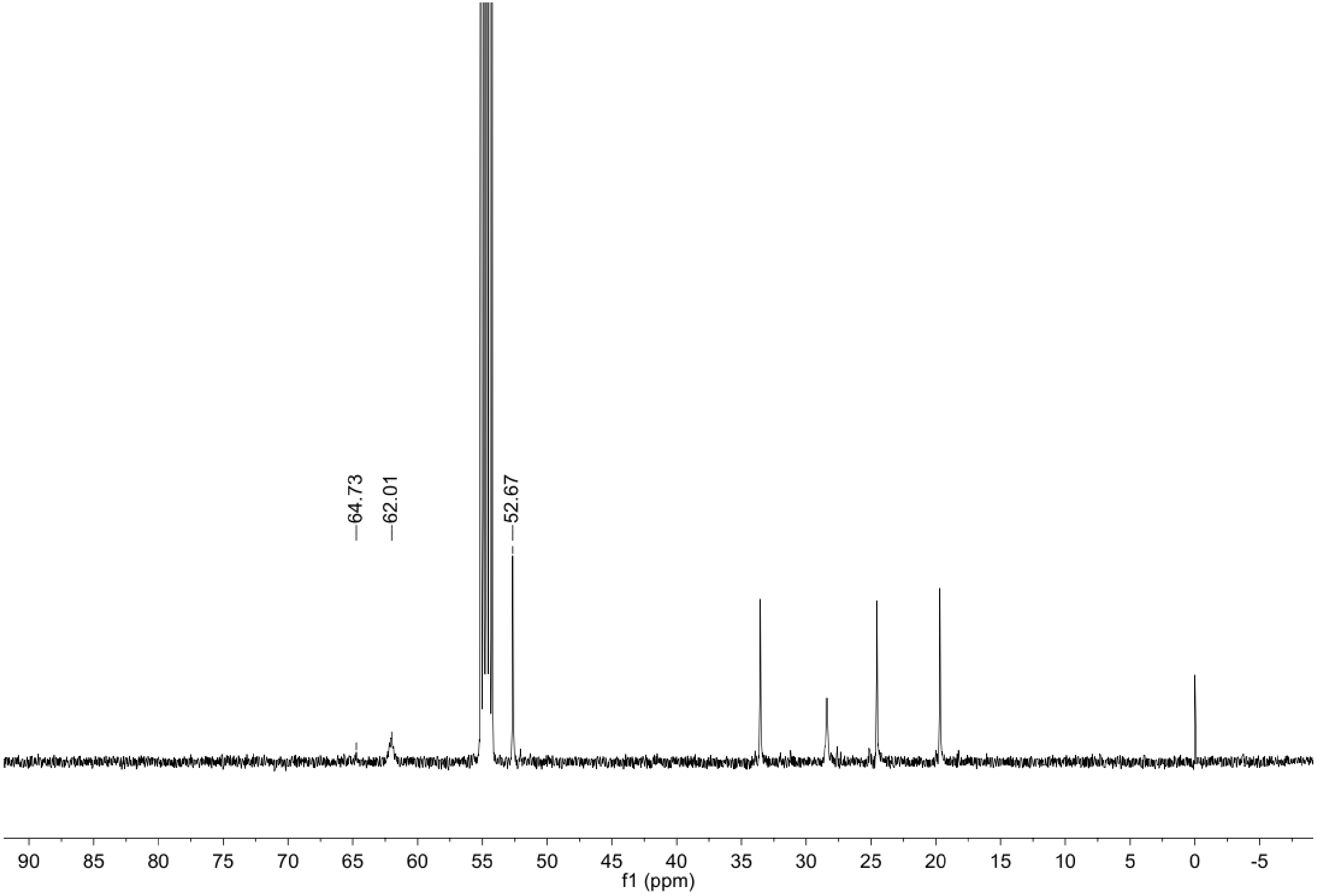

Figure A20. ^{1}H NMR spectra (500.13 MHz) of *cis*-2-bromocyclohexylamine in methanol-d_4 at −80 °C

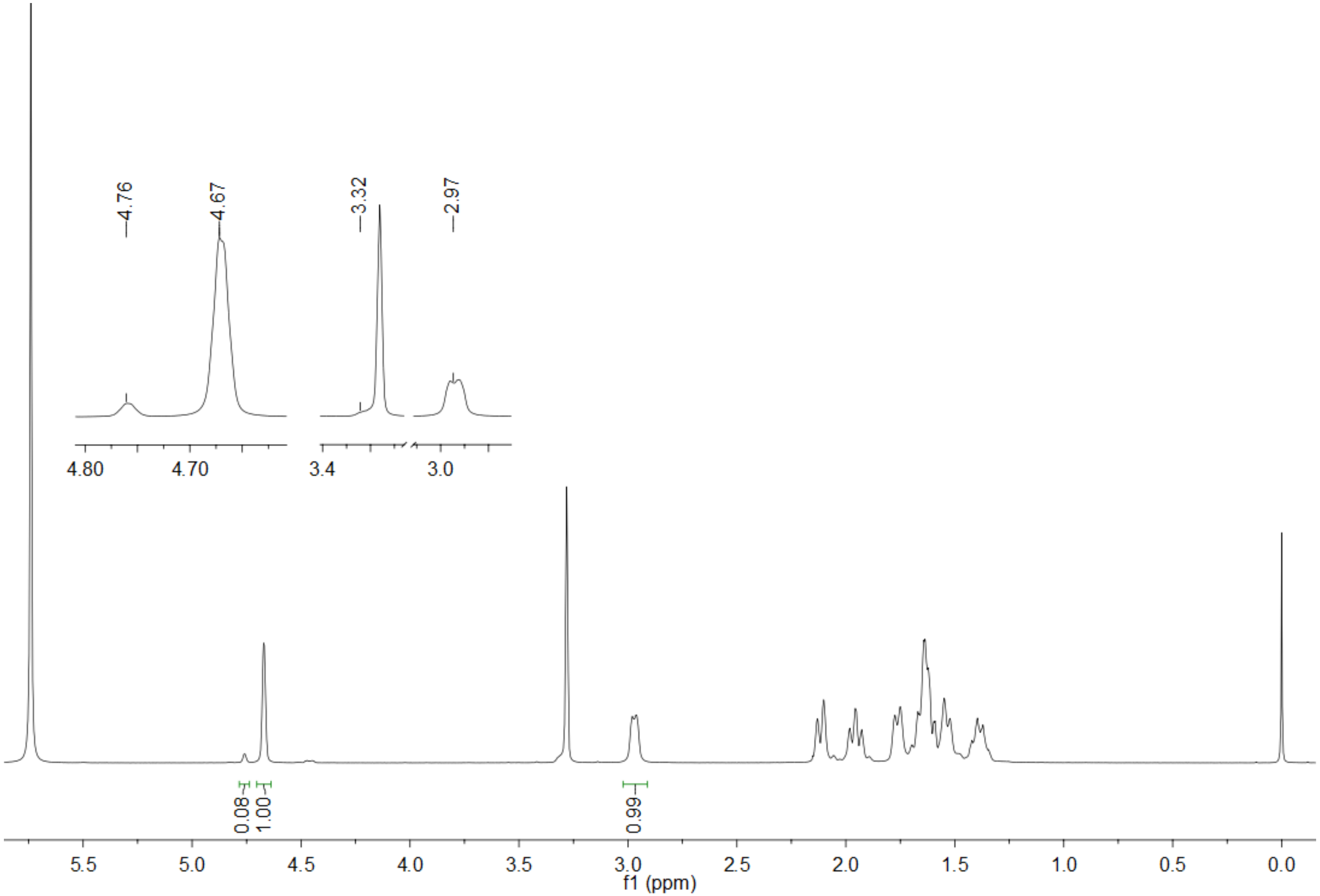

Figure A21. ^{13}C NMR spectra (125.77 MHz) of *cis*-2-bromocyclohexylamine in methanol-d_4 at −80 °C

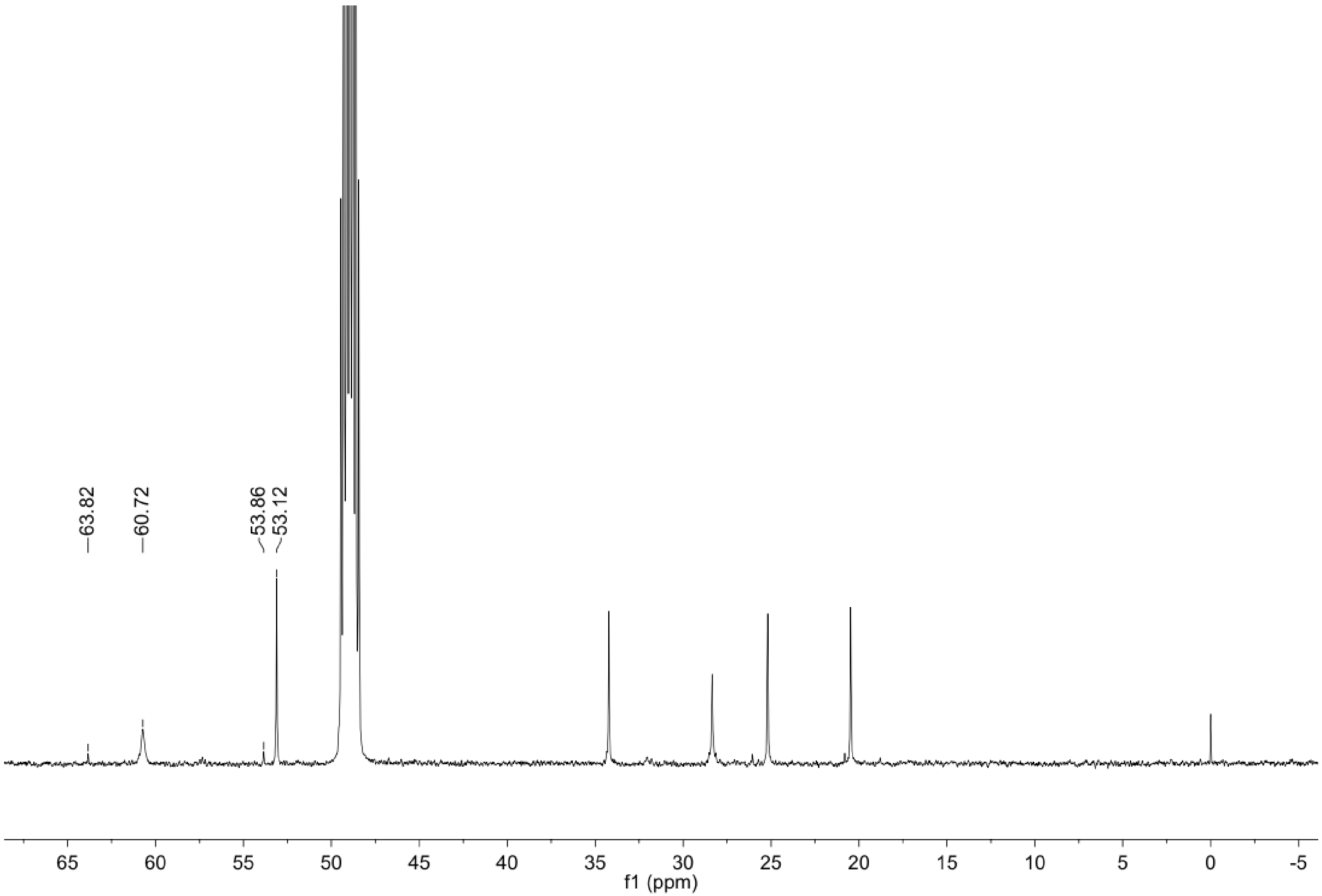

B. Theoretical calculations data

Table B1. ΔE values (kcal mol^{-1}) for all rotamers of *cis*-2-halocyclohexylamines in different theory levels in gas phase and in dichloromethane and methanol

			M06-2X / aug-cc-pVDZ			M06-2X / 6-311++G(2df, 2p)			MP2 / aug-cc-pVDZ			MP2/ 6-311++G(2df, 2p)		
	Conformer	Rotamer	Gas Phase	CH_2Cl_2	CH_3OH	Gas Phase	CH_2Cl_2	CH_3OH	Gas Phase	CH_2Cl_2	CH_3OH	Gas Phase	CH_2Cl_2	CH_3OH
**F**	**ae**	*a*	0.28	1.13	0.85	0.30	0.86	0.92	0.19	0.79	1.04	0.13	0.86	1.00
		*g*X	3.77	2.08	1.66	3.62	2.04	1.63	3.77	1.94	1.77	3.57	1.90	1.54
		*g*H	0.88	0.62	0.63	0.89	0.65	0.66	0.98	0.53	0.57	0.91	0.49	0.49
	**ea**	*a*	2.43	1.13	0.78	2.30	1.09	0.74	2.33	1.21	1.02	2.20	1.16	0.82
		*g*X	0.00	0.05	0.09	0.00	0.09	0.11	0.00	0.00	0.12	0.00	0.06	0.12
		*g*H	0.13	0.00	0.00	0.09	0.00	0.00	0.15	0.00	0.00	0.14	0.00	0.00
**Cl**	**ae**	*a*	0.38	1.07	3.80	0.42	1.15	1.19	0.13	0.89	1.13	0.13	0.91	1.05
		*g*X	3.57	2.08	4.36	3.53	2.23	1.85	3.66	2.19	2.01	3.53	2.18	1.85
		*g*H	0.76	0.64	3.34	0.77	0.73	0.74	0.77	0.56	0.61	0.79	0.55	0.56
	**ea**	*a*	2.44	1.25	3.52	2.26	1.23	0.85	2.29	1.41	1.20	2.14	1.30	0.98
		*g*X	0.04	0.23	2.87	0.04	1.18	0.19	0.02	0.15	0.25	0.00	0.14	0.18
		*g*H	0.00	0.00	0.00	0.00	0.00	0.00	0.00	0.00	0.00	0.00	0.00	0.00
**Br**	**ae**	*a*	0.44	1.08	1.07	0.45	1.06	1.10	0.33	1.14	1.33	0.10	0.89	1.03
		*g*X	3.75	2.43	2.05	3.78	2.49	2.14	3.81	2.49	2.24	3.39	2.10	1.78
		*g*H	0.91	0.87	0.86	0.95	0.93	0.94	0.98	0.80	0.82	0.74	0.50	0.51
	**ea**	*a*	2.35	1.18	0.78	2.29	1.25	0.89	2.21	1.41	1.18	2.00	1.22	0.92
		*g*X	0.12	0.09	0.08	0.13	0.12	0.13	0.06	0.21	0.31	0.04	0.22	0.26
		*g*H	0.00	0.00	0.00	0.00	0.00	0.00	0.00	0.00	0.00	0.00	0.00	0.00
**I**	**ae**	*a*	0.44	1.15	1.22	0.34	1.08	1.14	0.83	1.71	1.91	0.16	0.81	0.94
		*g*X	3.43	2.44	2.12	3.37	2.41	2.11	3.83	2.96	2.72	2.70	1.55	1.33
		*g*H	0.95	1.08	1.08	0.90	1.03	1.03	1.48	1.39	1.34	0.72	0.33	0.33
	**ea**	*a*	1.99	1.15	0.78	1.94	1.12	0.77	1.83	1.50	2.29	1.32	0.83	0.61
		*g*X	0.16	0.22	0.17	0.22	0.27	0.23	0.15	0.25	0.55	0.23	0.45	0.48
		*g*H	0.00	0.00	0.00	0.00	0.00	0.00	0.00	0.00	0.00	0.00	0.00	0.00

Table B2. ΔG^o values for **ea** conformer of *cis*-2-halocyclohexylamines calculated in different theory levels in gas phase and in dichloromethane and methanol

		ΔG^{oa}			
		M06-2X/ aug-cc-pVDZ	M06-2X/ 6-311++G(2df,2p)	MP2/ aug-cc-pVDZ	MP2/ 6-311++G(2df,2p)
Gas Phase	**F**	-0.39	-0.42	-0.29	-0.22
	**Cl**	-0.47	-0.50	-0.19	-0.22
	**Br**	-0.45	-0.48	-0.35	-0.16
Dichloromethane	**F**	-0.74	-0.71	-0.66	-0.73
	**Cl**	-0.55	-0.67	-0.58	-0.62
	**Br**	-0.80	-0.86	-0.79	-0.58
Methanol	**F**	-0.69	-0.72	-0.77	-0.73
	**Cl**	-0.59	-0.68	-0.62	-0.69
	**Br**	-0.77	-0.84	-0.79	-0.54

[a]Calculated by the weighted averages of both conformers

Table B3. Variation in total (ΔE_{total}), localized (ΔE_{loc}) and delocalization (ΔE_{deloc}) energies; total steric exchange (E_{steric}) and total electrostatic (E_{elect}) energies in kcal mol^{-1}, for all rotamers in **ae** and **ea** conformers of *cis*-2-halocyclohexylamines

	**ae**					**ea**				
	ΔE_{total}[a]	ΔE_{loc}[a]	ΔE_{deloc}[a]	E_{steric}[a]	E_{elect}[b]	ΔE_{total}[a]	ΔE_{loc}[a]	ΔE_{deloc}[a]	E_{steric}[a]	E_{elect}[b]
					Fluorine					
a	0.00	0.00	0.00	507.05	-0.50	2.38	1.02	3.41	504.31	0.77
***g*X**	3.52	7.19	10.71	507.91	0.57	0.00	0.00	0.00	509.24	-0.22
***g*H**	0.58	4.99	5.57	507.43	-0.61	0.12	1.33	1.45	508.26	-0.28
					Chlorine					
a	0.00	0.00	0.00	527.16	-0.13	2.48	0.63	0.64	526.78	0.67
***g*X**	3.58	9.77	6.68	527.51	0.38	0.01	0.00	0.00	526.92	0.21
***g*H**	0.49	6.47	5.12	530.23	-0.17	0.00	0.40	0.42	532.02	0.18
					Bromine					
a	0.00	0.00	0.00	534.05	-0.09	2.40	0.00	6.68	531.33	0.60
***g*X**	3.53	1.76	5.29	535.22	0.28	0.04	2.91	7.24	532.66	0.27
***g*H**	0.51	4.44	4.95	536.95	-0.12	0.00	2.62	6.91	535.73	0.25
					Iodine					
a	0.40	0.00	0.00	514.35	-0.04	2.08	0.00	0.00	512.51	0.61
***g*X**	3.61	1.53	4.74	515.85	0.29	0.19	2.73	0.84	514.24	0.29
***g*H**	0.88	3.95	4.43	517.85	-0.07	0.00	3.61	1.53	518.27	0.31

[a]M06-2X/6-311++G(2df,2p) in gas phase
[b]Calculated using the Amber force field GAFF. Positive values indicate more repulsive interactions

C. Principal component analysis data

A file containing the NBO values for PCA is available. The values correspond to the sum of all interactions involving the respective bonds.

R Script to generate PCA graphic:

```
head(NBO_total)
NBO <- NBO_total[,4:25]
head(NBO)
scale(NBO)
pc <- princomp(NBO, cor=F, score=T)
summary(pc)

library(factoextra)

# Generate the PCA graphical with the contribution vectors
fviz_pca_biplot(pc,
        # Individuals
        geom.ind = "point",
        fill.ind = NBO_total$Conf, col.ind = "black",
        pointshape = 21, pointsize = 4, labelsize = 6,
        palette = c("#56B4E9", "#000000"),
        # Variables
        col.var = "contrib", xlim = -25:23, ylim = -10:18,
        gradient.cols = c("#FFFFFF","#56B4E9","#56B4E9", "#000000","#000000"),
        title = NULL,
        legend.title = list(fill = "Conformer", color = "Contribution"))
```

D. Cartesian coordinates

Optimized conformers at the M06-2X/6-311++G(2df,2p) level in gas phase

cis-2-fluorocyclohexylamine, rotamer *a,* conformer **ae**

C	-0.46732500	0.94126600	0.40616900
C	-0.82492600	-0.53517000	0.49410500
C	0.00207300	-1.40441200	-0.42907700
C	1.48854500	-1.22215300	-0.11570600
C	1.88968600	0.24770300	-0.22601600
C	1.02697500	1.11421800	0.69094800
H	-1.03977600	1.46223200	1.18460500
H	-0.70858500	-0.86636800	1.53069400
H	-0.30363800	-2.44400800	-0.30754100
H	-0.20191000	-1.10829500	-1.45886800
H	1.69276200	-1.57856400	0.89948500
H	2.08747700	-1.83524000	-0.78929000
H	2.94243000	0.37363200	0.03011100
H	1.76162100	0.57877200	-1.25767300
H	1.21756600	0.84686500	1.73450200
H	1.28927300	2.16938100	0.58551400
N	-0.78330300	1.42395000	-0.93460100
H	-0.61138500	2.41874300	-1.00399700
H	-1.76235700	1.26632500	-1.13990200
F	-2.17450300	-0.67775900	0.17911500

E = -390,4328761 HF, ZeroPoint = 0,1809854 HF, NImag = 0

cis-2-fluorocyclohexylamine, rotamer *g*X, conformer **ae**

C	0.46319800	0.95967600	-0.37633500
C	0.86141900	-0.50967400	-0.47040700
C	0.04834400	-1.38279000	0.46488800
C	-1.43818200	-1.26232600	0.12303800
C	-1.89960800	0.19511800	0.15720500
C	-1.02697200	1.07045400	-0.74440200
H	1.05905100	1.50776600	-1.10866900
H	0.70620700	-0.84226000	-1.50277000
H	0.38947100	-2.41441900	0.37655000
H	0.23678700	-1.07014600	1.49535600
H	-1.60611900	-1.66567500	-0.88048100
H	-2.03207500	-1.86837300	0.80744200
H	-2.94324000	0.26912600	-0.15033700
H	-1.85910500	0.56496800	1.18615500
H	-1.16423600	0.75912900	-1.78466700
H	-1.33326300	2.11614200	-0.68149300
N	0.81230800	1.49665500	0.93371700
H	0.18957500	1.16521500	1.65855200
H	0.76230300	2.50645400	0.93129600
F	2.20658800	-0.65858400	-0.20076400

E = -390,4272646 HF, ZeroPoint = 0,1806654 HF, NImag = 0

cis-2-fluorocyclohexylamine, rotamer *g*H, conformer **ae**

C	0.47298300	0.95079600	-0.39833900
C	0.83027300	-0.53228600	-0.49632200
C	-0.00411100	-1.40481700	0.41826500
C	-1.49020700	-1.21682400	0.10651200
C	-1.89010100	0.25425800	0.21211000
C	-1.01786100	1.12386200	-0.69528100
H	1.05016900	1.47780900	-1.16145700
H	0.71502500	-0.86248100	-1.53398400
H	0.29836700	-2.44517200	0.29759900
H	0.20284800	-1.12922500	1.45578300
H	-1.69137900	-1.56917000	-0.91002000
H	-2.09282900	-1.83064800	0.77605900
H	-2.93970900	0.38085000	-0.05511800
H	-1.79940900	0.58431200	1.25101200
H	-1.20574800	0.84458300	-1.73571700
H	-1.27235500	2.17804200	-0.58863200
N	0.81422400	1.56073100	0.88062800
H	0.26703200	1.17145800	1.63757400
H	1.78851200	1.39172500	1.09936800
F	2.17489500	-0.68523800	-0.17539200

E = -390,4319473 HF, ZeroPoint = 0,1810079 HF, NImag = 0

cis-2-fluorocyclohexylamine, rotamer *a*, conformer **ea**

C	0.92946900	0.38353300	-0.46713500
C	0.34082600	-1.01445900	-0.31872800
C	-1.13385600	-1.04112100	-0.68104500
C	-1.91858200	-0.03635000	0.16225000
C	-1.33299400	1.36882500	0.02970700
C	0.15134600	1.37558000	0.39110700
H	0.77764800	0.65501500	-1.52397300
H	0.90997100	-1.72543900	-0.92578100
H	-1.51476200	-2.05270600	-0.53814200
H	-1.23418400	-0.80163300	-1.74329000
H	-1.87719600	-0.34653800	1.20867300
H	-2.96818300	-0.04358000	-0.13360400
H	-1.87708900	2.06598600	0.66764200
H	-1.45911100	1.71822800	-1.00011800
H	0.28901400	1.09782600	1.43835600
H	0.57514800	2.37415100	0.26160600
N	2.33284900	0.37768800	-0.08148800
H	2.72989000	1.30587100	-0.15034200
H	2.87981400	-0.23504300	-0.67340100
F	0.46909400	-1.43022300	0.99842800

E = -390,4294823 HF, ZeroPoint = 0,180779 HF, NImag = 0

cis-2-fluorocyclohexylamine, rotamer *g*X, conformer **ea**

C	0.91265400	0.41777700	-0.49583600
C	0.35793000	-0.99208300	-0.36716100
C	-1.12943600	-1.07054700	-0.64951700
C	-1.91792300	-0.08561100	0.21275900
C	-1.38160900	1.33497300	0.04492600
C	0.10915100	1.38828500	0.37538400
H	0.78513200	0.70312800	-1.54566800
H	0.93025300	-1.66839600	-1.00396900
H	-1.46598800	-2.09456500	-0.48389000
H	-1.28300800	-0.84593100	-1.70872400
H	-1.83248800	-0.38186700	1.26078500
H	-2.97597700	-0.13099000	-0.04695400
H	-1.93530500	2.02736200	0.67995000
H	-1.53633700	1.66122700	-0.98880500
H	0.26338500	1.11887200	1.42414600
H	0.50230400	2.39807200	0.24039000
N	2.34258400	0.37690500	-0.21931100
H	2.49749000	0.03335800	0.72177000
H	2.74528500	1.30406000	-0.27146100
F	0.57806200	-1.41327000	0.94825200

E = -390,433282 HF, ZeroPoint = 0,1809209 HF, NImag = 0

cis-2-fluorocyclohexylamine, rotamer *g*H, conformer **ea**

C	0.91175700	0.43505600	-0.49354500
C	0.37003800	-0.98709400	-0.35406000
C	-1.11436300	-1.08895400	-0.64765000
C	-1.91681600	-0.11135300	0.20927300
C	-1.39914400	1.31478100	0.03150900
C	0.09030700	1.40077000	0.35692700
H	0.79571800	0.71431200	-1.54615800
H	0.94639200	-1.66939200	-0.98541300
H	-1.43977700	-2.11701500	-0.48368900
H	-1.26444500	-0.86602500	-1.70795600
H	-1.82692600	-0.40120100	1.25869400
H	-2.97410600	-0.17303900	-0.05007200
H	-1.96113200	2.00285200	0.66382000
H	-1.56268000	1.63228300	-1.00354100
H	0.24868700	1.15280700	1.41079300
H	0.47394100	2.40885500	0.20331600
N	2.32222700	0.55808500	-0.15337100
H	2.89588500	-0.02018600	-0.75542500
H	2.47132100	0.23691500	0.79658300
F	0.58787300	-1.39855400	0.96199100

E = -390,4330975 HF, ZeroPoint = 0,1808752 HF, NImag = 0

cis-2-chlorocyclohexylamine, rotamer *a,* conformer **ae**

C	-0.05049500	1.04484400	-0.39152300
C	0.57489000	-0.34639200	-0.46192700
C	-0.09745200	-1.34734300	0.46088600
C	-1.58488700	-1.44595000	0.11193300
C	-2.25661400	-0.07649000	0.17932000
C	-1.54130300	0.92226800	-0.72895200
H	0.43417800	1.66170200	-1.15836500
H	0.52915500	-0.69498800	-1.49342000
H	0.39129000	-2.31637800	0.36218300
H	0.02327200	-1.01164900	1.49140300
H	-1.69389900	-1.85585500	-0.89753500
H	-2.07281800	-2.14644600	0.79034800
H	-3.30504000	-0.15432100	-0.11142100
H	-2.22819300	0.28767300	1.20739400
H	-1.63899400	0.60791500	-1.77198900
H	-2.00125200	1.91046300	-0.65478300
N	0.10118900	1.59548300	0.95009100
H	-0.31607500	2.51581000	1.00266600
H	1.08119500	1.68800500	1.18786300
Cl	2.34259000	-0.24505500	-0.09549400

E = -750,7928333 HF, ZeroPoint = 0,1797759 HF, NImag = 0

cis-2-chlorocyclohexylamine, rotamer *g*X, conformer **ae**

C	-0.05419900	1.05532100	-0.36171100
C	0.59949300	-0.32648900	-0.43797900
C	-0.07185900	-1.32479700	0.49094300
C	-1.54952300	-1.46580300	0.11539000
C	-2.26141700	-0.11352700	0.11369300
C	-1.52994500	0.89431500	-0.77345100
H	0.45016000	1.69945100	-1.08353400
H	0.52619900	-0.68358900	-1.46533600
H	0.43724700	-2.28562800	0.42283400
H	0.03668400	-0.98090800	1.52296100
H	-1.62054900	-1.90764800	-0.88330100
H	-2.04165900	-2.15694500	0.80043800
H	-3.29068500	-0.22880200	-0.22762100
H	-2.32338700	0.26693600	1.13748300
H	-1.57753200	0.55721200	-1.81338700
H	-2.01688700	1.87032600	-0.73074900
N	0.14451800	1.65652000	0.94943200
H	-0.03424100	2.65064200	0.92123400
H	-0.46416000	1.25641200	1.65070600
Cl	2.35952300	-0.23336600	-0.10465100

E = -750,7871298 HF, ZeroPoint = 0,1790293 HF, NImag = 0

cis-2-chlorocyclohexylamine, rotamer *g*H, conformer **ae**

C	-0.04842900	1.05851600	-0.38243600
C	0.57876700	-0.34242800	-0.46715100
C	-0.10020800	-1.34934700	0.44516800
C	-1.59081600	-1.43718300	0.10661400
C	-2.25701800	-0.06430200	0.16967300
C	-1.53494400	0.93463600	-0.73444800
H	0.43988800	1.68342900	-1.13240800
H	0.53080800	-0.68777600	-1.50024400
H	0.38194000	-2.32087700	0.34025100
H	0.03492200	-1.04017600	1.48498300
H	-1.70458000	-1.84431700	-0.90280200
H	-2.08036500	-2.13653700	0.78507700
H	-3.30401800	-0.13916200	-0.12599200
H	-2.25802800	0.29745800	1.20194300
H	-1.62732600	0.60378900	-1.77259900
H	-1.98702900	1.92336100	-0.66212000
N	0.10473000	1.73602300	0.89481200
H	-0.32899000	1.21984200	1.64926100
H	1.08448800	1.84131100	1.12747300
Cl	2.34124000	-0.25599200	-0.09300100

E = -750,79205 HF, ZeroPoint = 0,1795597 HF, NImag = 0

cis-2-chlorocyclohexylamine, rotamer *a*, conformer **ea**

C	-0.36129300	1.06559100	0.47191600
C	-0.49475800	-0.43062800	0.74806700
C	0.86790400	-1.06475700	1.00215400
C	1.85113800	-0.78842400	-0.13339300
C	1.97591400	0.71112200	-0.39738400
C	0.60862100	1.33467400	-0.67292800
H	0.09463100	1.46301600	1.39416100
H	-1.15390300	-0.58866700	1.60093800
H	0.74450800	-2.13512800	1.16343600
H	1.25767800	-0.63868600	1.93249900
H	1.50277900	-1.28945500	-1.03911500
H	2.82397600	-1.21437000	0.11419400
H	2.64545300	0.89271100	-1.23858200
H	2.42712600	1.19536500	0.47472800
H	0.17865500	0.92529700	-1.58950400
H	0.70065100	2.41410500	-0.81416000
N	-1.65403700	1.66478700	0.18814100
H	-1.57061100	2.66731000	0.08099200
H	-2.32201500	1.48564100	0.92771300
Cl	-1.31975400	-1.28212400	-0.61381600

E = -750,7892889 HF, ZeroPoint = 0,1791691 HF, NImag = 0

cis-2-chlorocyclohexylamine, rotamer *g*X, conformer **ea**

C	-0.35881800	1.06522600	0.48908500
C	-0.47283200	-0.42911000	0.77159900
C	0.88846700	-1.07631300	0.98215500
C	1.85301000	-0.78928300	-0.16649400
C	1.97802200	0.71319600	-0.41041100
C	0.60791500	1.34229900	-0.66505600
H	0.08095800	1.48293700	1.40322300
H	-1.12733200	-0.58370100	1.62538400
H	0.76359200	-2.14812200	1.13402100
H	1.29631000	-0.66309000	1.91074700
H	1.48772700	-1.27616600	-1.07350600
H	2.82819700	-1.22246800	0.05784200
H	2.64397400	0.90749800	-1.25181300
H	2.43307600	1.18465000	0.46688100
H	0.17869000	0.94139800	-1.58754800
H	0.69903300	2.42273800	-0.79567700
N	-1.68547800	1.64602900	0.35275200
H	-2.15769600	1.25098600	-0.45268600
H	-1.62324400	2.64590300	0.20917000
Cl	-1.33409000	-1.26005100	-0.59532800

E = -750,7932104 HF, ZeroPoint = 0,179558 HF, NImag = 0

cis-2-chlorocyclohexylamine, rotamer *g*H, conformer **ea**

C	-0.35901100	1.07458500	0.48553900
C	-0.47453600	-0.43108500	0.76444800
C	0.88662300	-1.07655100	0.98651300
C	1.85191300	-0.79155200	-0.16123500
C	1.97904200	0.71106500	-0.40229200
C	0.61494800	1.35186300	-0.65493200
H	0.07085000	1.49389400	1.40348900
H	-1.12612600	-0.59684700	1.62103900
H	0.76462300	-2.14768400	1.14581100
H	1.29105900	-0.65559800	1.91337900
H	1.48604400	-1.27757900	-1.06847600
H	2.82620200	-1.22663000	0.06338800
H	2.64339000	0.90462300	-1.24503700
H	2.43852600	1.17894700	0.47440500
H	0.18925700	0.96000400	-1.58366200
H	0.70317300	2.43160100	-0.77273400
N	-1.61648400	1.76043500	0.24742000
H	-2.26379100	1.61610600	1.01309800
H	-2.05796900	1.39085200	-0.58711900
Cl	-1.33198400	-1.26027600	-0.60104500

E = -750,7932344 HF, ZeroPoint = 0,1795122 HF, NImag = 0

cis-2-bromocyclohexylamine, rotamer *a*, conformer **ae**

C	-0.62731500	1.06670200	-0.38393700
C	0.05986800	-0.29438600	-0.44459900
C	-0.56947200	-1.32292300	0.47697000
C	-2.04648500	-1.49600000	0.10829800
C	-2.78643400	-0.16123600	0.15763500
C	-2.10634600	0.86767400	-0.74388000
H	-0.16577500	1.70741600	-1.14482000
H	0.05020200	-0.64719900	-1.47430300
H	-0.03659700	-2.26908400	0.39082600
H	-0.48125400	-0.97875700	1.50768800
H	-2.12137500	-1.91757700	-0.89938900
H	-2.50735400	-2.21551100	0.78582000
H	-3.82471400	-0.29191900	-0.14972300
H	-2.79285800	0.20868900	1.18389100
H	-2.17106900	0.54600200	-1.78707500
H	-2.61420400	1.83295400	-0.68099500
N	-0.52779300	1.62680800	0.95855000
H	-0.99752400	2.52207200	1.00163100
H	0.44163300	1.77622000	1.21176100
Br	1.98207300	-0.10342700	-0.04794500

E = -2864,76057 HF, ZeroPoint = 0,1790312 HF, NImag = 0

cis-2-bromocyclohexylamine, rotamer *g*X, conformer **ae**

C	-0.63206900	1.07643900	-0.35604700
C	0.08049000	-0.27514400	-0.42137400
C	-0.55265500	-1.30220000	0.50248900
C	-2.01875400	-1.51138300	0.10935300
C	-2.79149500	-0.19312200	0.09697400
C	-2.09530700	0.84378500	-0.78451500
H	-0.15481400	1.74379300	-1.07489600
H	0.04571000	-0.63651100	-1.44776000
H	-0.00261800	-2.24030500	0.44438900
H	-0.47601900	-0.95487300	1.53592700
H	-2.05758300	-1.95788600	-0.88889000
H	-2.48498500	-2.22346600	0.79111800
H	-3.81050500	-0.35500200	-0.25586500
H	-2.88153700	0.18712900	1.11865000
H	-2.11338200	0.50208000	-1.82379700
H	-2.62595900	1.79700700	-0.75203000
N	-0.47822000	1.69125700	0.95459000
H	-1.04302700	1.23658500	1.65944100
H	-0.74724000	2.66522800	0.92929200
Br	1.99309300	-0.09808100	-0.05140000

E = -2864,754941 HF, ZeroPoint = 0,1787064 HF, NImag = 0

cis-2-bromocyclohexylamine, rotamer *g*H, conformer **ae**

C	-0.62852600	1.08222800	-0.37477000
C	0.06468100	-0.28719700	-0.45099700
C	-0.56847500	-1.32288900	0.46088400
C	-2.04914100	-1.48933100	0.10439500
C	-2.78705400	-0.15272100	0.14851900
C	-2.10243000	0.87755700	-0.74926300
H	-0.16662400	1.73274500	-1.11915400
H	0.05110900	-0.63774300	-1.48216100
H	-0.03994800	-2.27085700	0.36891600
H	-0.46446700	-1.00333500	1.50085100
H	-2.12902900	-1.90968500	-0.90279700
H	-2.50910900	-2.20825800	0.78326800
H	-3.82346700	-0.28328000	-0.16445300
H	-2.82464500	0.21446200	1.17811000
H	-2.15980700	0.53848400	-1.78718200
H	-2.60459200	1.84264500	-0.68897400
N	-0.53398700	1.76917700	0.90251400
H	-0.93462800	1.22426800	1.65497300
H	0.43381300	1.94576100	1.14262400
Br	1.98099900	-0.10900900	-0.04669200

E = -2864,759763 HF, ZeroPoint = 0,1790238 HF, NImag = 0

cis-2-bromocyclohexylamine, rotamer *a*, conformer **ea**

C	0.76880400	1.20255500	0.44283300
C	-0.09020300	0.04312900	0.93824500
C	0.74223500	-1.21792800	1.13102200
C	1.53263600	-1.59046100	-0.12145300
C	2.39342100	-0.42027300	-0.59553100
C	1.54607200	0.83160400	-0.81513500
H	1.50362000	1.34024800	1.25478700
H	-0.59198000	0.31953400	1.86327200
H	0.09899300	-2.03776400	1.44758600
H	1.44028400	-1.01881700	1.95179000
H	0.83636800	-1.87596500	-0.91269700
H	2.15487300	-2.46114100	0.08803900
H	2.91598500	-0.68365100	-1.51542400
H	3.16256800	-0.21203000	0.15527300
H	0.83083500	0.67520600	-1.62541400
H	2.17718800	1.67602900	-1.10157900
N	-0.02587800	2.39504900	0.20823400
H	0.56518100	3.17993000	-0.03291300
H	-0.56861000	2.65288300	1.02331500
Br	-1.59148400	-0.32604500	-0.28381600

E = -2864,757269 HF, ZeroPoint = 0,1786596 HF, NImag = 0

cis-2-bromocyclohexylamine, rotamer *g*X, conformer **ea**

C	0.74235600	1.21614100	0.45555900
C	-0.07234900	0.02931900	0.95537700
C	0.77646200	-1.22254200	1.11442900
C	1.56716200	-1.56210900	-0.14713300
C	2.40165400	-0.36832600	-0.60803400
C	1.52701300	0.86889200	-0.81207200
H	1.47088200	1.39249800	1.25793600
H	-0.58031600	0.29769900	1.87656600
H	0.15039000	-2.05855100	1.42385400
H	1.47579700	-1.02284000	1.93427000
H	0.87355100	-1.85137900	-0.93958800
H	2.20786300	-2.42280800	0.04709700
H	2.93327700	-0.61048300	-1.52874800
H	3.16361900	-0.14974900	0.14738200
H	0.81949500	0.69866000	-1.62831200
H	2.13870200	1.72937900	-1.09126700
N	-0.09056500	2.40526000	0.37653500
H	-0.81169100	2.28124300	-0.32567900
H	0.46227100	3.20923000	0.10695300
Br	-1.58067600	-0.34565600	-0.27614200

E = -2864,761021 HF, ZeroPoint = 0,1789706 HF, NImag = 0

cis-2-bromocyclohexylamine, rotamer *g*H, conformer **ea**

C	0.73675400	1.23020300	0.45022200
C	-0.07484800	0.02696500	0.94974300
C	0.78834300	-1.21485000	1.11935900
C	1.58057000	-1.54977800	-0.14174600
C	2.40558400	-0.34930100	-0.60195100
C	1.53030500	0.88627100	-0.80573200
H	1.45778200	1.41853700	1.25658100
H	-0.58527700	0.27943200	1.87640700
H	0.17395800	-2.05671500	1.43654100
H	1.48711800	-1.00032600	1.93617300
H	0.88871700	-1.84535200	-0.93347500
H	2.22802900	-2.40533200	0.05285800
H	2.93608700	-0.58697600	-1.52439100
H	3.16843800	-0.12841700	0.15163800
H	0.82918300	0.71327000	-1.62773300
H	2.13250100	1.75370900	-1.07472400
N	-0.00888900	2.45847800	0.24988600
H	-0.53218400	2.71156900	1.07954300
H	-0.68170500	2.33688000	-0.49897600
Br	-1.57830500	-0.35933400	-0.27711500

E = -2864,761086 HF, ZeroPoint = 0,1788302 HF, NImag = 0

cis-2-iodocyclohexylamine, rotamer *a*, conformer **ae**

C	-1.07457200	1.06902500	-0.38200600
C	-0.36827000	-0.28298200	-0.43782100
C	-0.98662900	-1.31960200	0.48317700
C	-2.46029200	-1.51450500	0.10661100
C	-3.22031000	-0.19098600	0.14537200
C	-2.54832200	0.84391200	-0.75474900
H	-0.62206100	1.71793000	-1.14113500
H	-0.36559600	-0.63583900	-1.46710300
H	-0.44544200	-2.26167100	0.40322900
H	-0.91178000	-0.97480700	1.51492100
H	-2.52229400	-1.94120600	-0.89973700
H	-2.91415700	-2.23787800	0.78508700
H	-4.25410300	-0.33812800	-0.16999800
H	-3.24103900	0.18233200	1.17028200
H	-2.59640800	0.51549900	-1.79655900
H	-3.07166200	1.80177100	-0.70262800
N	-1.00342900	1.63563400	0.95943200
H	-1.53202300	2.49744900	1.00551800
H	-0.04615700	1.84995800	1.21262200
I	1.76409600	-0.06139700	-0.03007600

E = -301,9275635 HF, ZeroPoint = 0,1785411 HF, NImag = 0

cis-2-iodocyclohexylamine, rotamer *g*X, conformer **ae**

C	-1.07460200	1.07633900	-0.35875500
C	-0.35172500	-0.26910200	-0.42416100
C	-0.97784100	-1.30633800	0.49483400
C	-2.44487800	-1.52161800	0.10394100
C	-3.22487000	-0.20817400	0.09993200
C	-2.53871900	0.83321600	-0.78282700
H	-0.60907400	1.74766700	-1.08141200
H	-0.37152100	-0.62841700	-1.45124400
H	-0.42789000	-2.24441000	0.43173300
H	-0.90468400	-0.96878000	1.53207900
H	-2.48309600	-1.96334300	-0.89645600
H	-2.90448400	-2.23979800	0.78416700
H	-4.24528600	-0.37433900	-0.24709800
H	-3.31017400	0.16948300	1.12307900
H	-2.55658100	0.49224300	-1.82227500
H	-3.07469600	1.78364100	-0.74821100
N	-0.92427700	1.69675900	0.94904600
H	-1.46306900	1.22350300	1.66211400
H	-1.22577200	2.66129400	0.92797200
I	1.76834100	-0.05966000	-0.03123700

E = -301,9224549 HF, ZeroPoint = 0,1782584 HF, NImag = 0

cis-2-iodocyclohexylamine, rotamer *g*H, conformer **ae**

C	-1.07614900	1.08496300	-0.37152700
C	-0.36394100	-0.27546900	-0.44436600
C	-0.98597900	-1.31910200	0.46745100
C	-2.46355400	-1.50706500	0.10302300
C	-3.22101800	-0.18133400	0.13615500
C	-2.54457400	0.85460300	-0.76042900
H	-0.62282100	1.74509600	-1.11228400
H	-0.36776600	-0.62667300	-1.47494200
H	-0.44934300	-2.26314400	0.38181500
H	-0.89537600	-0.99937700	1.50886800
H	-2.53051700	-1.93306900	-0.90266900
H	-2.91669400	-2.22948000	0.78305200
H	-4.25309800	-0.32831300	-0.18401800
H	-3.27170800	0.19054800	1.16349300
H	-2.58508900	0.50962700	-1.79708700
H	-3.06159700	1.81232400	-0.70945900
N	-1.01216200	1.77592400	0.90530300
H	-1.38611700	1.21494200	1.65963600
H	-0.05543300	2.00417700	1.14646800
I	1.76249000	-0.06542800	-0.02984600

E = -301,9268035 HF, ZeroPoint = 0,1786654 HF, NImag = 0

cis-2-iodocyclohexylamine, rotamer *a*, conformer **ea**

C	1.27839100	1.16716400	0.41478500
C	0.35648200	0.10321000	0.99986400
C	1.07756200	-1.22977300	1.16010900
C	1.74872600	-1.69355900	-0.13029900
C	2.68077000	-0.61541400	-0.68181100
C	1.93861700	0.70509000	-0.87905800
H	2.07549500	1.25191300	1.17454500
H	-0.04400700	0.44102700	1.95294200
H	0.38899700	-1.98351700	1.53891700
H	1.84683500	-1.08419500	1.92777300
H	0.98197500	-1.92731500	-0.87264500
H	2.30157400	-2.61433800	0.05845300
H	3.11907100	-0.94202300	-1.62520900
H	3.51012300	-0.46483400	0.01661900
H	1.16551000	0.60040200	-1.64381500
H	2.62548300	1.48231700	-1.22210100
N	0.58689300	2.42649100	0.20462700
H	1.23282800	3.14796400	-0.08836900
H	0.12194700	2.74651600	1.04529100
I	-1.47013900	-0.15584300	-0.16974200

E = -301,9248944 HF, ZeroPoint = 0,1784137 HF, NImag = 0

cis-2-iodocyclohexylamine, rotamer *g*X, conformer **ea**

C	1.27104100	1.17564400	0.42027500
C	0.36950900	0.09939800	1.01287100
C	1.08829200	-1.23439400	1.15180900
C	1.75335800	-1.68847400	-0.14527300
C	2.67682300	-0.60340800	-0.69698200
C	1.92987200	0.71766400	-0.88314700
H	2.06846500	1.28168100	1.16893700
H	-0.03579500	0.44519600	1.95840600
H	0.40633800	-1.99328000	1.53281300
H	1.86232800	-1.08893800	1.91514600
H	0.98357200	-1.92366500	-0.88411000
H	2.31204700	-2.60726200	0.03562800
H	3.11571600	-0.92504800	-1.64199900
H	3.50679600	-0.44994800	0.00034100
H	1.15971000	0.60940000	-1.65236300
H	2.61320400	1.49884800	-1.22281400
N	0.58592700	2.45716100	0.36881500
H	-0.16949700	2.43115800	-0.30738900
H	1.22437200	3.18864600	0.08272800
I	-1.46570000	-0.15972600	-0.16461000

E = -301,9279012 HF, ZeroPoint = 0,1786834 HF, NImag = 0

cis-2-iodocyclohexylamine, rotamer *g*H, conformer **ea**

C	1.26691100	1.19018000	0.41754300
C	0.36641800	0.09648200	1.00851500
C	1.10148500	-1.22875800	1.15483100
C	1.76828400	-1.67604700	-0.14307400
C	2.68348100	-0.58254200	-0.69167700
C	1.93532600	0.73698700	-0.87554700
H	2.05834700	1.30565600	1.17120200
H	-0.04235200	0.42494500	1.96105700
H	0.43067400	-1.99475500	1.54141700
H	1.87620900	-1.07057200	1.91508700
H	0.99941900	-1.91574000	-0.88152100
H	2.33384500	-2.59105300	0.03577700
H	3.12205500	-0.89805400	-1.63879400
H	3.51410400	-0.42778000	0.00425400
H	1.17034900	0.62627200	-1.65056300
H	2.61067200	1.52705000	-1.20320100
N	0.66464000	2.49745700	0.24045100
H	0.20207400	2.80933000	1.08600000
H	-0.03962200	2.46562700	-0.48839300
I	-1.46452200	-0.16907400	-0.16526500

E = -301,928203 HF, ZeroPoint = 0,1786371 HF, NImag = 0
